# Deriving neighborhood-level diet and physical activity measurements from anonymized mobile phone location data for enhancing obesity estimation

**DOI:** 10.1186/s12942-022-00321-4

**Published:** 2022-12-30

**Authors:** Ryan Zhenqi Zhou, Yingjie Hu, Jill N. Tirabassi, Yue Ma, Zhen Xu

**Affiliations:** 1 GeoAI Lab, Department of Geography, University at Buffalo, The State University of New York, Buffalo, NY 14260 USA; 2 Jacobs School of Medicine and Biomedical Sciences, University at Buffalo, The State University of New York, Buffalo, NY 14260 USA; 3 College of Landscape Architecture, Nanjing Forestry University, Nanjing, Jiangsu 210037 China

**Keywords:** Obesity, Diet, Physical activity, Anonymized mobile phone location data, GeoAI

## Abstract

**Background:**

Obesity is a serious public health problem. Existing research has shown a strong association between obesity and an individual’s diet and physical activity. If we extend such an association to the neighborhood level, information about the diet and physical activity of the residents of a neighborhood may improve the estimate of neighborhood-level obesity prevalence and help identify the neighborhoods that are more likely to suffer from obesity. However, it is challenging to measure neighborhood-level diet and physical activity through surveys and interviews, especially for a large geographic area.

**Methods:**

We propose a method for deriving neighborhood-level diet and physical activity measurements from anonymized mobile phone location data, and examine the extent to which the derived measurements can enhance obesity estimation, in addition to the socioeconomic and demographic variables typically used in the literature. We conduct case studies in three different U.S. cities, which are New York City, Los Angeles, and Buffalo, using anonymized mobile phone location data from the company SafeGraph. We employ five different statistical and machine learning models to test the potential enhancement brought by the derived measurements for obesity estimation.

**Results:**

We find that it is feasible to derive neighborhood-level diet and physical activity measurements from anonymized mobile phone location data. The derived measurements provide only a small enhancement for obesity estimation, compared with using a comprehensive set of socioeconomic and demographic variables. However, using these derived measurements alone can achieve a moderate accuracy for obesity estimation, and they may provide a stronger enhancement when comprehensive socioeconomic and demographic data are not available (e.g., in some developing countries). From a methodological perspective, spatially explicit models overall perform better than non-spatial models for neighborhood-level obesity estimation.

**Conclusions:**

Our proposed method can be used for deriving neighborhood-level diet and physical activity measurements from anonymized mobile phone data. The derived measurements can enhance obesity estimation, and can be especially useful when comprehensive socioeconomic and demographic data are not available. In addition, these derived measurements can be used to study obesity-related health behaviors, such as visit frequency of neighborhood residents to fast-food restaurants, and to identify primary places contributing to obesity-related issues.

**Supplementary Information:**

The online version contains supplementary material available at 10.1186/s12942-022-00321-4.

## Background

Obesity is a serious public health problem. In the United States, nearly 42.4% of the adult population are considered overweight or obese [1], and the estimated annual medical cost of obesity ranges from $147 billion to nearly $210 billion per year [2, 3]. Obesity can increase the risk of various health issues, including heart disease, type 2 diabetes, sleep apnea, depressive disorder, and others [4, 5]. Given its substantial costs to individuals and the society, reducing obesity is a critical task for public health policymakers and related organizations.

Existing research has shown a strong association between obesity and an individual’s diet and physical activity [6–9]. If we extend such an association to the neighborhood level, information about the diet and physical activity of the residents of a neighborhood may improve the estimate of neighborhood-level obesity prevalence and help identify the neighborhoods that are more likely to suffer from high prevalence of obesity. This has important meaning as neighborhood environments, both physical and social environments, are known to affect the health behaviors of neighborhood residents [10–14]. Accordingly, the ability to more accurately identify neighborhoods with high obesity prevalence allows intervention and prevention programs to focus on these neighborhoods and mitigate their obesity issues by, for example, improving their built environment and enhancing social support in these communities [15, 16].

Researchers have examined a variety of neighborhood-level factors and their associations with obesity-related outcomes [17, 18]. These factors include race/ethnicity composition, percentages of different age groups, percentages of different educational levels, median income, unemployment rate, poverty level, median home value, median home age (i.e., median year since built), and population density [19–21]. Neighborhood-level variables representing diet and physical activity of the neighborhood residents are much rarer, and studies that examined related factors typically focused on availability or access, such as the availability or proximity to fast food outlets and greenspace in or near neighborhoods [22–26]. The results of these studies, however, are mixed: some studies found significant associations between obesity and these availability based variables, whereas some other studies reported primarily null associations [14, 23, 25, 27, 28]. The fact that a fast-food restaurant or a greenspace is available in or near a neighborhood does not necessarily mean that the neighborhood residents will consume fast food in such a restaurant or engage in physical activity in that greenspace.

One possible reason that variables measuring the health behaviors of neighborhood residents on diet and physical activity have been rarely used is that it is challenging to collect data. Compared with variables representing availability or access which can be calculated based on the locations of places (e.g., fast-food restaurants) and neighborhood boundaries, data about the health behaviors of residents typically need to be collected via surveys and interviews. Conducting these surveys and interviews, however, requires considerable financial and labor resources. Even when those required resources are available, completing such surveys can take a long time. These resource and time requirements can become more difficult to manage when we need to collect health behavior data related to diet and physical activity for large geographic areas, such as the three different cities studied in this work.

Since the COVID-19 pandemic, there has been an increasing use of anonymized mobile phone location data in health studies [29, 30]. This type of data provides new opportunities for deriving measurements on the health behaviors of neighborhood residents related to diet and physical activity. These mobile phone location data are mainly collected from applications installed on smartphones, such as navigation, weather, and social media applications [31–33]. Data companies, such as SafeGraph (whose data are used in this study), collected data from many mobile phone applications and then aggregated data to geographic areas (e.g., census tracts) and places visited by people, which are typically referred to as points-of-interest (POIs) in the literature [34, 35]. The data are anonymized and are not associated with any personal identifying information. In addition, because the data were aggregated to geographic areas and POIs, they do not contain any individual-level movement trajectories. While having these limitations for good privacy protection reasons, these anonymized mobile phone location data do provide valuable information about how people living in a geographic area visit surrounding POIs. Among these POIs, there are places linked to diet and physical activity, such as fast-food restaurants, fitness centers, and nature parks.

The objective of this study is twofold. First, we propose a method for deriving neighborhood-level measurements on diet and physical activity of neighborhood residents from anonymized mobile phone location data and related POIs. Second, we investigate the research question: *To what extent can the diet and physical activity measurements derived from anonymized mobile phone location data improve obesity estimation at the neighborhood level?* We conduct case studies in three different US cities and employ five different statistical and machine learning models to examine and understand the potential enhancement brought by the derived diet and physical activity measurements for obesity estimation.

This study addresses one important objective of the thematic issue “New horizons in geospatial lifestyle and food environment research”, i.e., using smart technologies and big geospatial data to obtain accurate and precise measurements related to overweight, obesity (OO) and type-2 diabetes (T2D) [36]. Instead of relying on the proximity of a neighborhood to fast-food restaurants, fitness centers, or nature parks, we derive measurements on how neighborhood residents actually visit these places based on anonymized mobile phone location data. This approach avoids making the assumption that people tend to visit the fast-food restaurants closest to their neighborhoods, and enables us to use more precise and accurate measurements to study OO and T2D. The remainder of this paper is organized as follows. “Methods” section describes the study area and data, and presents our designed analyses and method for deriving neighborhood-level diet and physical activity measurements. “Results” section presents the analysis results and “Discussion” section discusses the results and implications. Finally, “Conclusions” section concludes this work.

## Methods

### Study area and data

#### Study area

**Fig. 1:**
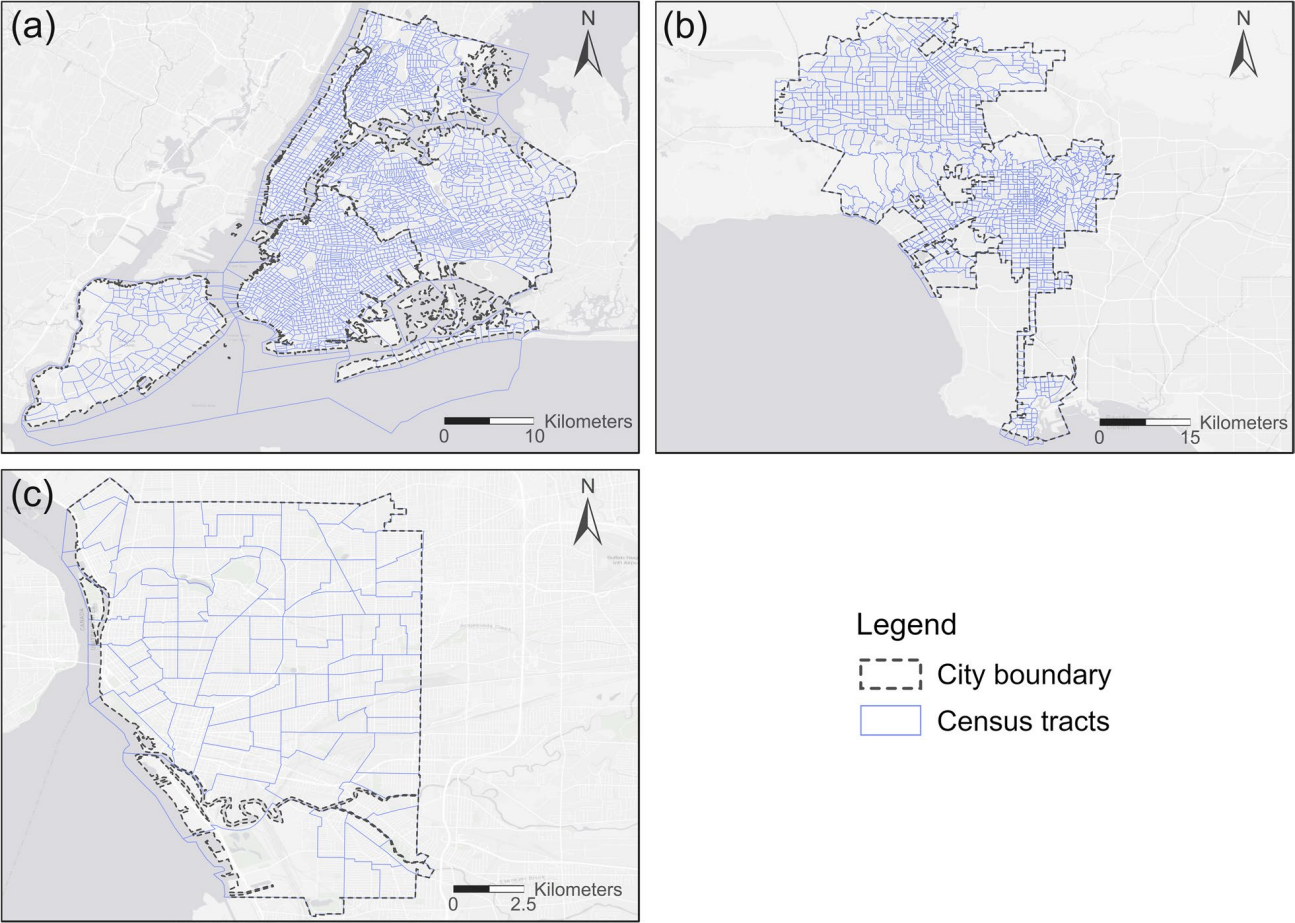
The city boundaries of NYC, LA, and Buffalo and their census tracts: **a** NYC; **b** LA; **c** Buffalo

We selected three US cities for this study, which are New York City (NYC), Los Angeles (LA), and Buffalo. We chose these three cities because NYC and LA are two megacities located on the east coast and west coast respectively, while Buffalo is a medium-sized city that the authors are familiar with and it is located close to the Midwest region of the US. Although other cities could also be selected for this study, these three cities allow a comparison of the results from cities located in different geographic regions and of different sizes. The time period of our study is the year 2018, and the geographic unit of analysis is census tract which is roughly comparable to neighborhoods. We choose this time period and this geographic unit largely because of data availability: the obesity data used in this study is from the *PLACES* project of the Centers for Disease Control and Prevention (CDC), whose data is in the year 2018 and the smallest geographic unit is census tract [37]. Figure 1 shows the city boundaries of NYC, LA, and Buffalo and their census tracts. The geographical boundaries of these three cities were obtained from the 2018 TIGER/line Shapefile products provided by the US Census Bureau.

#### Obesity data

The outcome variable that we focus on in this study is neighborhood-level obesity prevalence. We obtained the census tract-level obesity prevalence among adults (age ≥18) data from the CDC PLACES Project, and the obesity prevalence is recorded in percentages (e.g., a value of 26.6 indicates the obesity prevalence for that census tract is 26.6%). Among all the census tracts in the three studied cities, 227 census tracts (7.0%) were excluded from this study, because they either have fewer than 50 residents or their obesity prevalence is missing from the CDC data. The total number of census tracts included for analysis for NYC, LA, and Buffalo are 1995, 947, and 77, respectively. Note that there are only 77 census tracts in Buffalo, and this small number of geographic units affects our analysis results and training of machine learning models later. We will also compute global Moran’s I index for obesity prevalence. Global Moran’s I is a common metric for quantifying spatial autocorrelation in data, and it is calculated based on both locations and values (e.g., obesity prevalence) at these locations. The value of global Moran’s I ranges between [− 1, 1], with − 1 indicating a strong negative spatial autocorrelation (i.e., different values tend to cluster together) and 1 indicating a strong positive spatial autocorrelation (i.e., similar values tend to cluster together).

#### Anonymized mobile phone location data

**Fig. 2:**
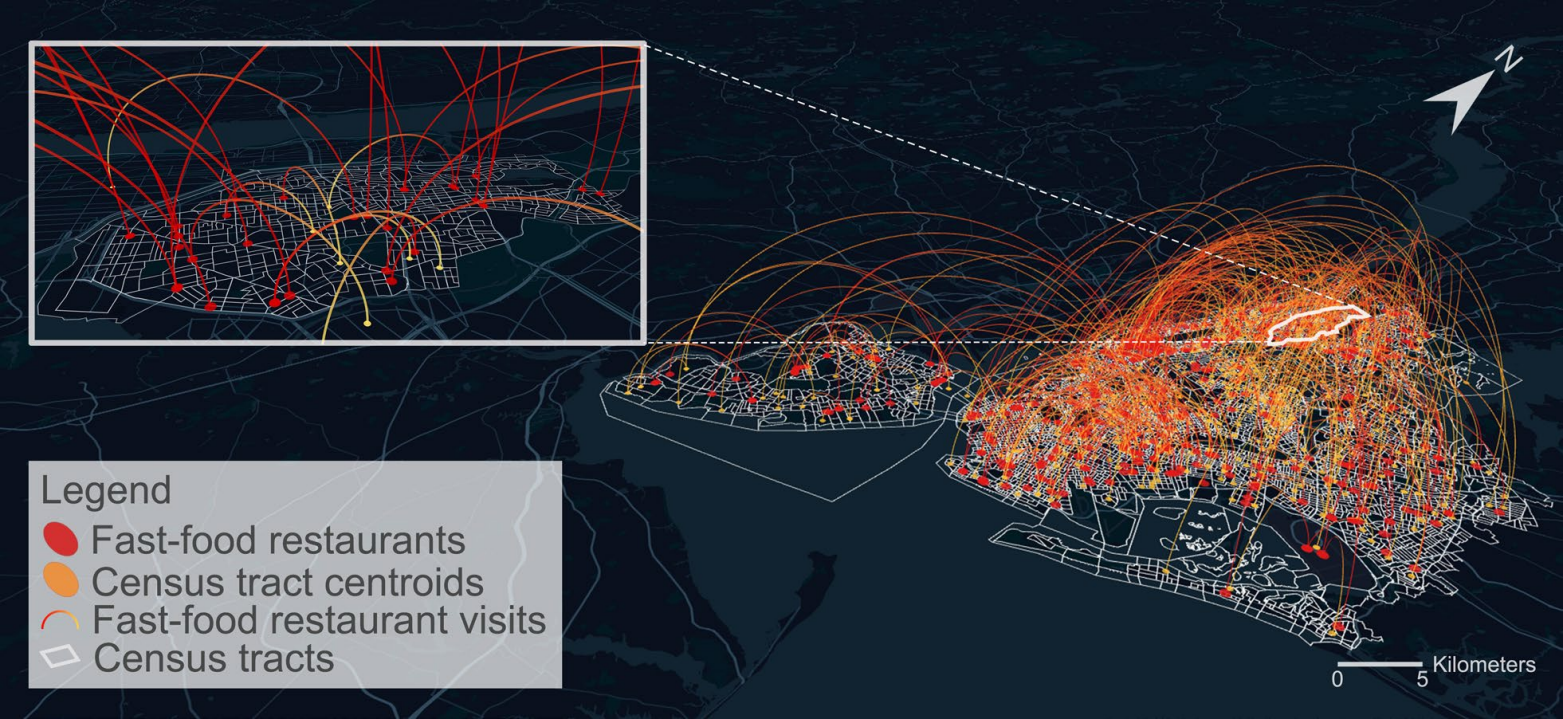
A map visualization of the visits from census tracts to fast-food restaurants in a week of 2018 in NYC

The anonymized mobile phone location data used in this study are provided by the company SafeGraph, which opened their data for the research community for free. The data of SafeGraph were collected from over 45 million smart mobile devices (mostly smartphones) and roughly 11.8 million POIs covering the entire United States [38, 39]. As noted previously, the data were aggregated to census tracts and POIs, and we only have POI visits without individual-level GPS trajectories. Using a sample of data in NYC, we plot out the visits from census tracts to fast-food restaurants in a week of 2018 (Fig. 2). In this figure, each curve links a census tract (whose centroid is represented by a yellow dot) and a fast-food restaurant (represented by a red dot), which indicates some residents from the census tract visited that fast-food restaurant during that week.

#### Neighborhood-level socioeconomic and demographic data

In this study, we aim to understand to what extent the neighborhood-level diet and physical activity measurements derived from anonymized mobile phone location data can enhance obesity estimation, in addition to the neighborhood-level socioeconomic and demographic variables typically used in existing studies. We select variables in six categories: (1) race and ethnicity, (2) gender, marital status, and age, (3) education, (4) economic status, (5) housing condition, and (6) urbanicity. These variables are selected based on the existing literature. In particular, variables in categories (1), (2), (3), (4), (6) were used in previous studies, such as Ball et al. in 2002 [40], Black et al. in 2008 [17], Yan et al. in 2015 [24], and Puciato et al. in 2020 [41], and variables in category (5) were used in previous studies, such as Norman et al. in 2010 [42] and Fitzpatrick et al. in 2018 [20]. Table 1 presents the detailed notations and descriptions of these variables. We obtained data for these variables from the American Community Survey (ACS) of the US Census Bureau. Note that there is a potential limitation in the socioeconomic and demographic data from the Census and the obesity prevalence data from CDC. The estimates of these two datasets are interval estimates, and the quality of the data varies spatially as pointed out in the literature [43]. Nevertheless, these datasets are the best we can have for this study, and we acknowledge their limitations.

**Table 1:** Notations and descriptions of the six categories of neighborhood-level variables

Variable notations	Descriptions
(1) Race and ethnicity	
% White	Percentage of population in White
% Black	Percentage of population in Black or African American
% Ame Indi and AK Native	Percentage of population in American Indian and Alaska Native
% Asian	Percentage of population in Asian
% Nati Hawa and Paci Island	Percentage of population in Native Hawaiian and Other Pacific Islander
% Hispanic or Latino	Percentage of Hispanic or Latino population
(2) Gender, marital status, and age	
% male	Percentage of male population
% married	Percentage of married population age 15 or over
% age 18–29	Percentage of population between age 18 to 29
% age 30–39	Percentage of population between age 30 to 39
% age 40–49	Percentage of population between age 40 to 49
% age 50–59	Percentage of population between age 50 to 59
% age ≥60	Percentage of population equal and over age 60
(3) Education	
% < highschool	Percentage of population age 25 or over without high school completion
% ≥highschool < university	Percentage of population age 25 or over with high school completion and without bachelor degree
%≥ university	Percentage of population age 25 or over with bachelor degree or higher degree
(4) Economic status	
Med income	Median household income
% unemployment	Percentage of unemployed labor force population age 16 or over
% below poverty line	Percentage of population below poverty line
% food stamp/SNAP	Percentage of households received food stamp/supplemental nutrition assistance program (SNAP) in the past 12 months
(5) Housing condition	
Median value units built	Median value of the house units built (in dollars)
Median year units built	Median year of the house units built
% renter-occupied housing units	Percentage of renter-occupied housing units
(6) Urbanicity	
Population density	Population density (people per square kilometer)

We do not include *% age* < *18* in category (2), because the obesity data from CDC do not include population below 18 years old

### Overview of study design

**Fig. 3:**
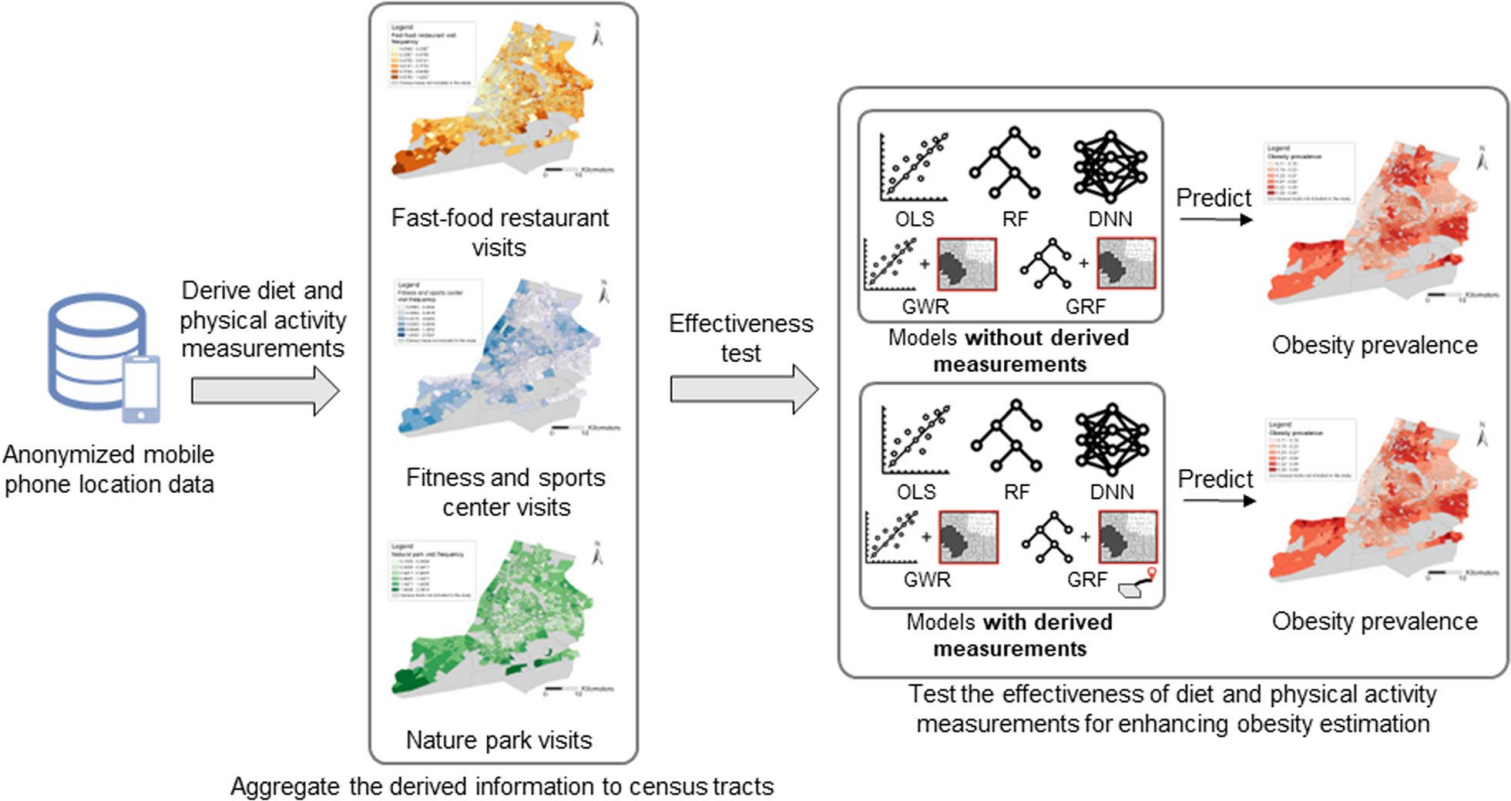
An overview of the study design using NYC as an example

The objective of this study is to derive neighborhood-level diet and physical activity measurements from anonymized mobile phone location data and investigate to what extent the derived measurements can enhance obesity estimation. Figure 3 provides an overview of our study design, using NYC as an example. We first derive neighborhood-level diet and physical activity measurements from anonymized mobile phone location data based on the visits of neighborhood residents to different types of POIs. In particular, we focus on three types of POIs, which are fast-food restaurants, fitness and sports centers, and nature parks. We will explain why we choose to focus on these three types of POIs in the next section. With the derived measurements, we conduct two sets of analyses to examine their ability to enhance obesity estimation at the neighborhood level. In the first set of analyses (baseline analyses), we estimate obesity prevalence at the neighborhood level using the six categories of socioeconomic and demographic variables (see Table 1); in the second set of analyses (test analyses), we add the derived diet and physical activity measurements to the socioeconomic and demographic variables to examine the extent to which these derived measurements can help improve obesity estimation. We use five different statistical and machine learning models to perform these two sets of analyses.

### Deriving neighborhood-level diet and physical activity measurements

The neighborhood-level diet and physical activity measurements are derived in the following three steps. First, we identify a number of POI types that are shown to be linked to diet and physical activity based on the literature. In particular, three types of POIs are identified in this study, which are fast-food restaurants [26, 44], fitness and sports centers [45, 46], and nature parks [47, 48]. It is worth noting that these three types of POIs only capture some aspects of the everyday life of people related to diet and physical activity, and they certainly do not represent all the places where people can do exercise or purchase healthy food. For example, people can purchase healthy food also from grocery stores and full-service restaurants. However, these places can serve unhealthy food as well [36]. Meanwhile, the anonymized mobile phone location data do not contain information about the specific products that a person purchased at a place. Thus, we do not know, e.g., whether a grocery store or full-service restaurant visit also involves healthy food purchase or not. By contrast, visits to fast-food restaurants, fitness and sports centers, and nature parks have relatively clear associations with corresponding diet and physical activity. Thus, we eventually chose to focus on these three types of POIs.

**Fig. 4:**
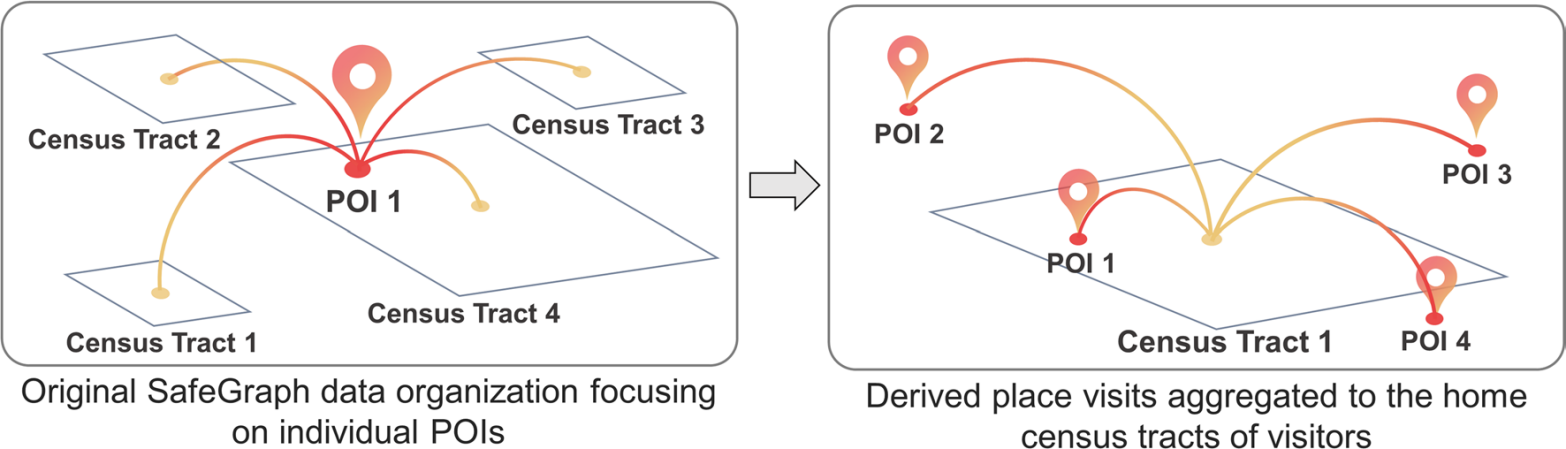
An illustration of deriving neighborhood-level diet and physical activity measurements by reversing the data focus from POIs to census tracts. The POIs visited by the residents of a neighborhood can be outside of the neighborhood or even outside of the city boundary

Second, we utilize the anonymized mobile phone location data to derive total number of visitors from the studied census tracts to these three types of POIs. The original SafeGraph data are organized focusing on POIs by providing information about the number of people who have visited these POIs during a time period and the home census tracts of the POI visitors (inferred based on the nighttime locations of the mobile devices in the previous six weeks). Here, we reverse the focus of the data from POIs to census tracts and compute the total number of visitors from each census tract who visited a type of POIs. In this way, we can measure how the residents of neighborhoods (approximated by census tracts) visit different POIs. Figure 4 illustrates this process. It is worth noting that the residents of a neighborhood can visit POIs outside of their neighborhood and also outside of the studied city boundary (especially in the case of LA whose boundary has a narrow strip connecting to the southern parts of the city). When deriving POI visit information for a neighborhood, we included all POIs that were visited by the neighborhood residents regardless of whether the POIs are within the neighborhood or city boundary. The total numbers of POIs used to derive neighborhood-level visit information are provided in the Additional file 1: Table S1. There is a privacy related limitation in the data: SafeGraph recorded the number of visitors from a census tract to a POI as 4 if the actual number of visitors equals or is smaller than 4 for privacy protection. Thus, a census tract that has 4 visitors to a POI recorded in the data may in fact have 2, 3 or 4 visitors (if a census tract has only 1 visitor to a POI, this visit is removed by SafeGraph for privacy protection). To address this data limitation, we generate randomized numbers from 2 to 4 following a power-law distribution typically observed in human travel behaviors [49, 50].

Third, we divide the total number of visitors aggregated to census tracts by the total number of devices residing in the same census tracts to obtain *place visit frequency.* Eq. (1) summarizes this computing process:

1 $$\mathrm{Place\,visit\,frequency\,for\,census\,tract_j}=\frac{\sum_{i=1}^{n}{V}_{ij}}{{S}_{j}}$$

where v_ij_ is the number of visitors from census tract_j_ to a POI_i_ related to diet and physical activity; *n* is the total number of POIs in one type of places (e.g., fast-food restaurants) in the study area; ${S}_{j}$ is the total number of mobile devices in census tract_j_. We apply Eq. (1) to each census tract and to each of the three types of POIs. As a result, we obtain three types of diet and physical activity measurements.

### Statistical and machine learning models

We use five different statistical and machine learning models to examine the potential improvement brought by the derived measurements for obesity estimation. These models are: ordinary least squares (OLS), geographically weighted regression (GWR), random forest (RF), deep neural network (DNN), and geographical random forests (GRF). The former two are statistical models while the latter three are machine learning or artificial intelligence (AI) models. We use machine learning models instead of only statistical models alone because there has been an increasing interest in using AI models for health studies [51–53]. AI models are often based on mechanisms quite different from statistical models, such as neurons and decision trees. Thus, using both statistical and machine learning models allows us to understand how the derived diet and physical activity measurements can function in models with different internal mechanisms. Among the five models, GWR and GRF are spatially explicit models that accommodate spatial heterogeneity typically existing in geographic data [54, 55], while OLS, RF and DNN are non-spatial models. In the following, we briefly describe each model.

#### Ordinary least squares

OLS is a statistical model of analysis that estimates the relationship between multiple input independent variables and the target outcome variable. The OLS model used in this work is in the form of Eq. (2):

2 $$\mathrm{Obesity\,prevalence}=\,{\theta }_{0} +{\theta }_{r}r +{\theta }_{a}a +{\theta }_{s}s+{\theta }_{e}e+{\theta }_{h}h {+\theta }_{u}u (+{\theta }_{v}v)+\varepsilon$$

where $\theta$_r_, $\theta$_a_, $\theta$_s_, $\theta$_e_, $\theta$_h_ $,\theta$_u_ are the coefficients for the six categories of socioeconomic and demographic variables respectively, and $\theta$_v_ are the coefficients for the three types of diet and physical activity measurements based on place visits. $\theta$_v_v is within a pair of parentheses in the equation because diet and physical activity measurements will not be included in the baseline analyses. Note that each of $\theta$_r_, $\theta$_a_, $\theta$_s_, $\theta$_e_, $\theta$_h_ $,\theta$_u_, $\theta$_v_ contains multiple coefficients for the variables in that category (e.g., $\theta$_v_ contains three regression coefficients for the three types of diet and physical activity measurements).

#### Geographically weighted regression

GWR has been frequently used in geographic data analysis to model spatially varying relationships between variables [56, 57]. GWR fits a local OLS model for each geographic unit (i.e., census tract in this study) based on weighted data from nearby geographic units, and therefore can be considered as an ensemble of local models [58]. Specifically, the GWR model used in this work is in the form of Eq. (3):

3 $${\mathrm{Obesity\,prevalence }\,=\theta }_{0}({x}_{i}, {y}_{i}) +{\theta }_{r}({x}_{i}, {y}_{i})r +{\theta }_{a}({x}_{i}, {y}_{i})a +{\theta }_{s}({x}_{i}, {y}_{i})s+{\theta }_{e}({x}_{i}, {y}_{i})e+{\theta }_{h}\left({x}_{i}, {y}_{i}\right)h {+ \theta }_{u}\left({x}_{i}, {y}_{i}\right)u \left(+{\theta }_{v}\left({x}_{i}, {y}_{i}\right)v\right)+{\varepsilon }_{i}$$

where ($x$_i_, $y$_i_) is the spatial coordinates of the geographic unit *i*. The coefficients have the same meaning as used in OLS, but will vary across different geographic locations capturing the potentially heterogenous local processes. We configured the GWR model following the recommendations of the GWR developers [59]: we employed the bisquare kernel to specify the weights of the data from nearby geographic units based on their distances to the current location, and we applied the golden section search approach to identify the optimal bandwidth which determines the number of nearby geographic units to be included for fitting the local model.

#### Random forest

Random forest is a bagging-based machine learning model that applies an ensemble learning technique by constructing a group of decision trees [60]. Compared with OLS that assumes a linear relation, RF can model nonlinear relations between input features and the target variable. Given this ability, RF has been used in a variety of previous studies in which the input features and the target variable likely have a nonlinear relation [61, 62].

#### Deep neural network

DNNs and other deep learning models have shown outstanding predictive power in recent years [63, 64]. A DNN is made of multiple successive layers of neurons and can learn a complex nonlinear relation between the input features and the target variables. The model architecture can be configured flexibly with different numbers of total layers and different numbers of neurons. Additional components, such as dropout layers or batch normalization, could also be added depending on the application.

#### Geographical random forests

GRF is a disaggregation of a global RF model into multiple local RF models across different spatial locations [55]. The core idea of GRF is similar to GWR, in which a local RF model is fitted for each geographic unit. This means that for each location *i*, a local RF is trained but is based on only a number of nearby geographic units. Such a design allows the RF model to adapt to different local contexts.

For all the models, we implement them using Python and related packages: *statsmodels* for OLS, *mgwr* for GWR, *scikit-learn* for RF, *tensorflow* for DNN, and *scikit-learn* for GRF*.* For machine learning models, we also perform hyperparameter tuning to identify the best model architecture. Two metrics, R^2^ and root mean square error (RMSE), are utilized for assessing the accuracy of the five models for obesity estimation. For the statistical models, their R^2^ and RMSE are directly obtained from the model fitting results. For the machine learning models, their R^2^ and RMSE are obtained via a tenfold cross-validation process. In addition, for the two statistical models, OLS and GWR, we also report their adjusted R^2^ and Akaike information criterion (AIC) which take into account the increased model complexity when additional variables, i.e., the derived diet and physical activity measurements, are included. Given that GWR is an ensemble of local linear models, its AIC is calculated based on the log-likelihood of the full model and the effective number of parameters derived based on the selected bandwidth. We used the *mgwr* package from the GWR developers to calculate its AIC values, and more details can be seen in their papers [59, 65].

## Results

### Neighborhood-level obesity prevalence and derived diet and physical activity measurements

**Fig. 5:**
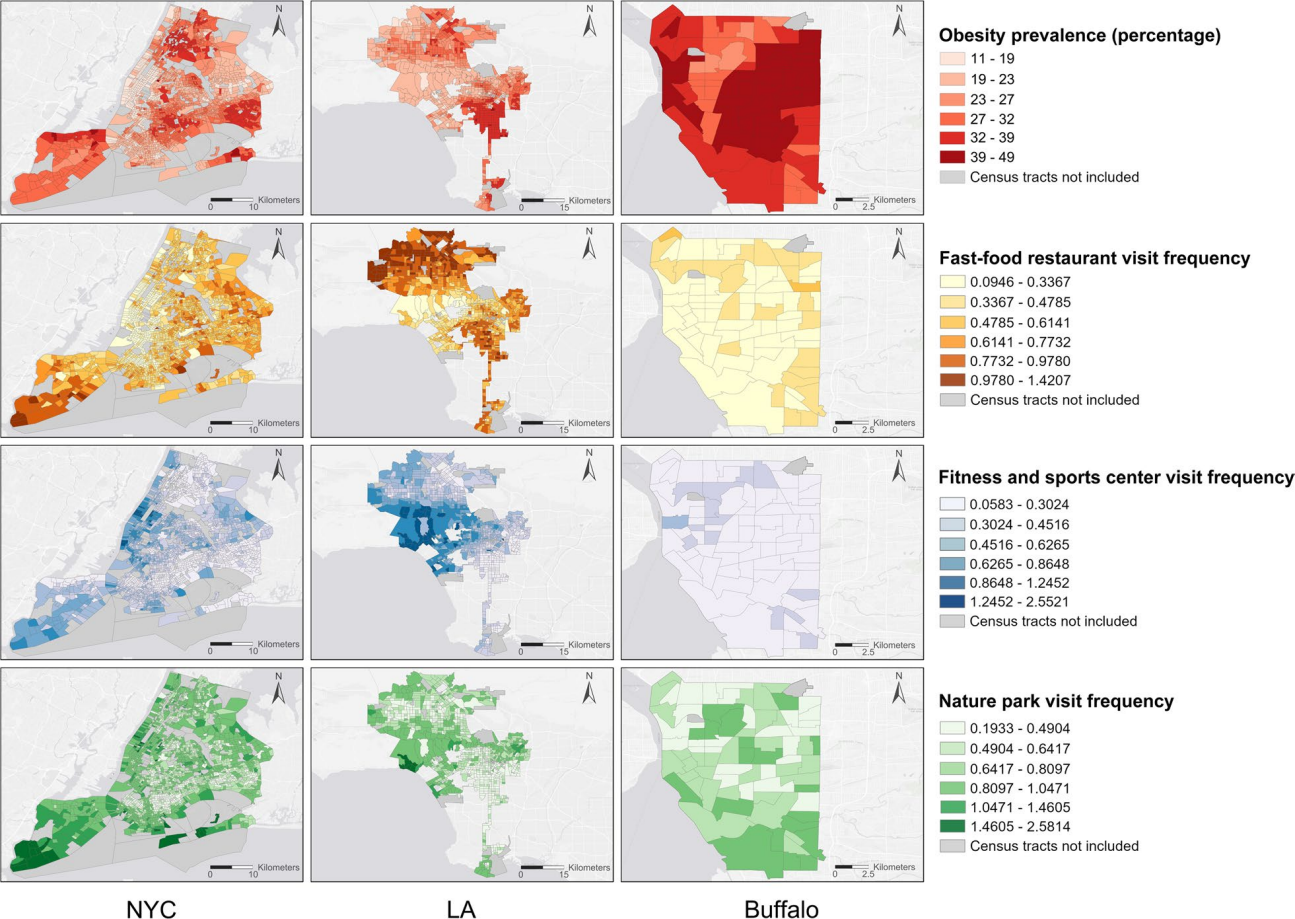
Neighborhood-level obesity prevalence and the three derived diet and physical activity measurements for NYC, LA, and Buffalo

The obesity prevalence at the census tract level in NYC, LA, and Buffalo in the year of 2018 from the *PLACES* project are visualized as the first row (the top row) in Fig. 5. The three diet and physical activity measurements derived from the anonymized mobile phone location data are visualized in the second (fast-food restaurant visit frequency), third (fitness and sports center visit frequency), and fourth row (nature park visit frequency) in Fig. 5.

As can be seen, census tracts with high obesity prevalence in NYC tend to be clustered in the northern area (Bronx), the south of Queens, Brooklyn, and the north of Staten Island; in LA, high obesity prevalence tend to be clustered in the northern and southern areas of the city; and in Buffalo, high obesity prevalence tend to be clustered on the east side of the city, and we know that this region consists of mostly low-income neighborhoods. Given the observed clusters, we compute the global Moran’s *I* index to examine the existence of spatial autocorrelation in obesity prevalence. The results show that the obesity prevalence in NYC, LA, and Buffalo all have statistically significant and positive spatial autocorrelations, with Moran’s *I* indexes 0.740 (p < 0.001), 0.741 (p < 0.001), and 0.668 (p < 0.001), respectively. These results suggest that in all three cities neighborhoods with high obesity prevalence tend to be clustered together rather than being distributed more randomly within a city.

By further looking into the three derived diet and physical activity measurements (the second to the fourth row in Fig. 5), we can see interesting geographic patterns. In particular, the fast-food restaurant visit frequencies in NYC and LA are largely consistent with their corresponding obesity prevalence patterns, i.e., census tracts with higher obesity prevalence tend to have higher fast-food restaurant visit frequency. Meanwhile, the fitness and sports center visit frequencies in NYC and LA show largely inverted patterns compared with their obesity prevalence patterns, i.e., census tracts with higher obesity prevalence tend to have lower fitness and sports center visit frequency. For nature park visit frequencies, they show a similar inverted pattern as the fitness and sports center visit frequencies in NYC and LA, i.e., census tracts with higher obesity prevalence tend to have lower nature park visit frequencies. In Buffalo, this similarity and difference in distribution patterns seem to be weaker, but we do observe that census tracts with higher obesity prevalence tend to have slightly higher fast-food restaurant visit frequency and census tracts with lower obesity prevalence tend to have slightly higher fitness and sports center visit frequency and slightly higher nature park visit frequency.

### Multicollinearity diagnosis

Before examining the ability of the derived diet and physical activity measurements to enhance obesity estimation, we first carry out a series of diagnostic tests to examine whether there exists multicollinearity among the neighborhood-level socioeconomic and demographic variables. To do so, we compute the variance inflation factor (VIF) for the 24 variables, and all variables are standardized by their mean and standard deviation before the analyses. We then gradually remove the variables with the highest VIF values until they are all smaller than the typical cut-off value 5. Table 2 shows the results of these VIF tests.

**Table 2:** VIF values obtained from the multicollinearity tests

Variable	First test	Second test	Final test
(1) Race and ethnicity			
% White	58.265	58.261	–
% Black	49.264	49.26	2.754
% Ame Indi and AK Native	1.091	1.091	1.06
% Asian	19.277	19.273	2.026
% Nati Hawa and Paci Island	1.035	1.032	1.02
% Hispanic or Latino	44.031	44.031	4.729
(2) Gender, marital status, and age			
% male	1.333	1.332	1.317
% married	4.26	4.26	4.133
% age 18–29	4.314	4.314	4.216
% age 30–39	3.402	3.401	3.219
% age 40–49	1.792	1.792	1.765
% age 50–59	1.763	1.763	1.762
% age > = 60	3.574	3.574	3.347
(3) Education			
% < highschool	> 1000	6.079	4.946
% ≥highschool < university	> 1000	-	-
% ≥university	> 1000	8.586	-
(4) Economic status			
Median income	5.934	5.934	4.501
% unemployment	1.448	1.448	1.433
% below poverty line	4.395	4.395	4.309
% food stamp/SNAP	3.95	3.95	3.843
(5) Housing condition			
Median value units built	2.356	2.354	2.258
Median year units built	1.181	1.181	1.173
% renter-occupied housing units	4.644	4.642	4.466
(6) Urbanicity			
Population density	1.647	1.647	1.603

As can be seen, the first test shows that three variables have extremely high VIF values (% < highschool, % >  = highschool < university, and % >  = university), suggesting severe multicollinearity for these variables. This is likely due to the fact that the variables in the education category are composition measures that add up to 1. Accordingly, in the second test, we remove the “% >  = highschool < university” variable in the education category. The result of the second test shows substantially reduced VIF values but some of these values are still larger than 5. In the final test, we further remove two variables with high VIF values in two categories, which are “% White” in the race and ethnicity category and “% >  = university” in the education category. After these two variables are removed, the final test shows that the VIF values of all variables are smaller than 5, suggesting low multicollinearity among them. We therefore use these remaining 21 socioeconomic and demographic variables in the following analyses.

### Results from the five statistical and machine learning models

One main objective of this study is to understand to what extent the diet and physical activity measurements derived from the anonymized mobile phone location data can help enhance obesity estimation at the neighborhood level. To achieve this objective, we perform two sets of analyses in three different cities using five different statistical and machine learning models. In the baseline analyses, we use the 21 socioeconomic and demographic variables (identified through the multicollinearity tests) as the independent variables; in the test analyses, we use the three diet and physical activity measurements in addition to the 21 variables. Table 3 summarizes the obtained results, with the three cities as the three main rows (i.e., NYC, LA, and Buffalo) and the five statistical and machine learning models as the five main columns (i.e., OLS, GWR, RF, DNN, and GRF).

**Table 3:** A summary of the results for testing the effectiveness of the derived diet and physical activity measurements for enhancing obesity estimation using five statistical and machine learning models (i.e., OLS, GWR, RF, DNN, and GRF)

		OLS		GWR		RF		DNN		GRF	
City	Fit measures	Base line	Test	Base line	Test	Base line	Test	Base line	Test	Base line	Test
NYC	R^2^	0.861	**0.869**	0.975	**0.977**	0.894	**0.898**	0.879	**0.895**	0.934	0.934
	RMSE	2.194	**2.127**	0.926	**0.898**	1.916	**1.881**	2.045	**1.907**	1.508	**1.506**
	adjusted R^2^	0.860	**0.868**	0.968	**0.969**	–	–	–	–	–	–
	AIC	8840.7	**8723.0**	6244.3	**6237.8**	–	–	–	–	–	–
LA	R^2^	0.963	**0.964**	0.972	**0.974**	0.950	0.950	0.924	0.912	0.951	0.951
	RMSE	1.043	**1.034**	0.903	**0.872**	1.213	**1.210**	1.495	1.613	1.204	1.208
	adjusted R^2^	0.962	**0.963**	0.968	**0.970**	–	–	–	–	–	–
	AIC	2811.5	**2800.7**	2732.3	**2696.6**	–	–	–	–	–	–
Buffalo	R^2^	0.976	0.976	0.982	**0.983**	0.869	**0.873**	–	–	0.877	0.875
	RMSE	1.088	**1.079**	0.934	**0.914**	2.514	**2.478**	–	–	2.444	2.456
	adjusted R^2^	0.966	0.965	0.969	0.968	–	–	–	–	–	–
	AIC	275.4	280.1	272.6	276.8	–	–	–	–	–	–

Adjusted R^2^ and AIC can only be calculated for the two statistical models; DNN model cannot be trained for Buffalo due to the small number of data records (only 77 data records)

Numbers in bold indicate improvements over the baseline analyses

As can be seen in Table 3, adding the three diet and physical activity measurements to the input of the models increases the accuracy of obesity prevalence estimation in most of the analyses, as demonstrated by the higher R^2^ and lower RMSE values in the test analyses. Note that we do not highlight the performance values when there is a tie between the test and baseline analyses in order to provide a more conservative view of the results, although most of these tied test analyses have slightly better performance values than the baseline analyses beyond the third digit. This improvement is overall consistent even when we take into consideration model complexity as demonstrated by the adjusted R^2^ and AIC values, and is overall consistent across the five different models even though these models have different inner mechanisms. However, the improvement is small compared with using the 21 neighborhood-level socioeconomic and demographic variables for obesity estimation. We will further discuss this result in the Discussion section.

### Regression coefficients and feature importance

We present the regression coefficients output by the OLS model and the feature importance output by the RF model in order to understand the roles played by different independent variables in estimating neighborhood-level obesity prevalence. Table 4 shows the regression coefficients from the OLS model.

**Table 4:** Regression coefficients obtained via the OLS model in NYC, LA, and Buffalo

Variables	NYC	LA	Buffalo
(1) Race and ethnicity			
% Black	2.5460***	1.366***	3.5307***
% Ame Indi and AK Native	– 0.0550	0.0371	0.0154
% Asian	– 1.5253***	– 1.5574***	– 0.0841
% Nati Hawa and Paci Island	– 0.0200	– 0.0263	– 0.0763
% Hispanic or Latino	1.1223***	1.3375***	– 0.0823
(2) Gender, marital status, and age			
% male	0.0611	0.0269	0.1607
% married	0.0632	0.0549	– 0.4479
% age 18–29	– 0.5314***	– 0.7602***	– 1.7860***
% age 30–39	– 0.1871*	– 0.1909**	0.2533
% age 40–49	0.0983	– 0.0605	0.1242
% age 50–59	0.1825**	0.0852	– 0.1283
% age > = 60	– 0.7922***	– 0.1804*	– 0.5141
(3) Education			
% < highschool	0.4386***	0.7150***	0.5131
(4) Economic status			
Med income	– 0.8246***	– 0.3493***	– 0.8983*
% unemployment	0.0858	0.0894*	0.1544
% below poverty line	1.1520***	1.2396***	1.4296***
% food stamp/SNAP	0.4041***	0.5628***	0.3141
(5) Housing condition			
Median value units built	– 0.3446***	– 0.1955**	– 0.8326*
Median year units built	0.2663***	– 0.0964*	0.0784
% renter-occupied housing units	– 0.8679***	0.2952***	0.1380
(6) Urbanicity			
Population density	– 0.4485***	0.0247	– 0.7433***
(7) Three derived diet and physical activity measurements			
Fast-food restaurant visit frequency (vf)	0.6903***	0.1797***	– 0.1673
Fitness and sports center vf	– 0.2361**	– 0.1660*	0.0294
Nature park vf	– 0.0436	– 0.0048	– 0.0037

*p-value < 0.05; **p-value < 0.01; ***p-value < 0.001

As can be seen, variables related to poverty level, such as *median income*, *% below poverty line*, and *median value units built*, all show statistically significant associations with neighborhood-level obesity prevalence across the three different cities. Variables related to racial and ethnic composition, in particular *% Black*, also show significant associations with obesity prevalence. Racial and ethnic variables, however, are often intertwined with socioeconomic status. Overall, NYC and LA share more similarity in the obtained regression coefficients compared with those in Buffalo. For example, the variables of *%* < *highschool*, *% food stamp/SNAP*, and *% renter-occupied housing units*, all show statistically significant associations with obesity prevalence in NYC and LA but not in the city of Buffalo. For the three diet and physical activity measurements, *fast-food restaurant vf* shows significant and positive associations (*p* < 0.001) with the obesity prevalence in both NYC and LA, and *fitness and sports center vf* shows significant and negative associations (*p* < 0.05 and *p* < 0.01) also in NYC and LA. *Nature park vf* does not show a significant association with obesity prevalence in all three cities, and the three variables do not show significant associations with obesity in Buffalo.

**Fig. 6:**
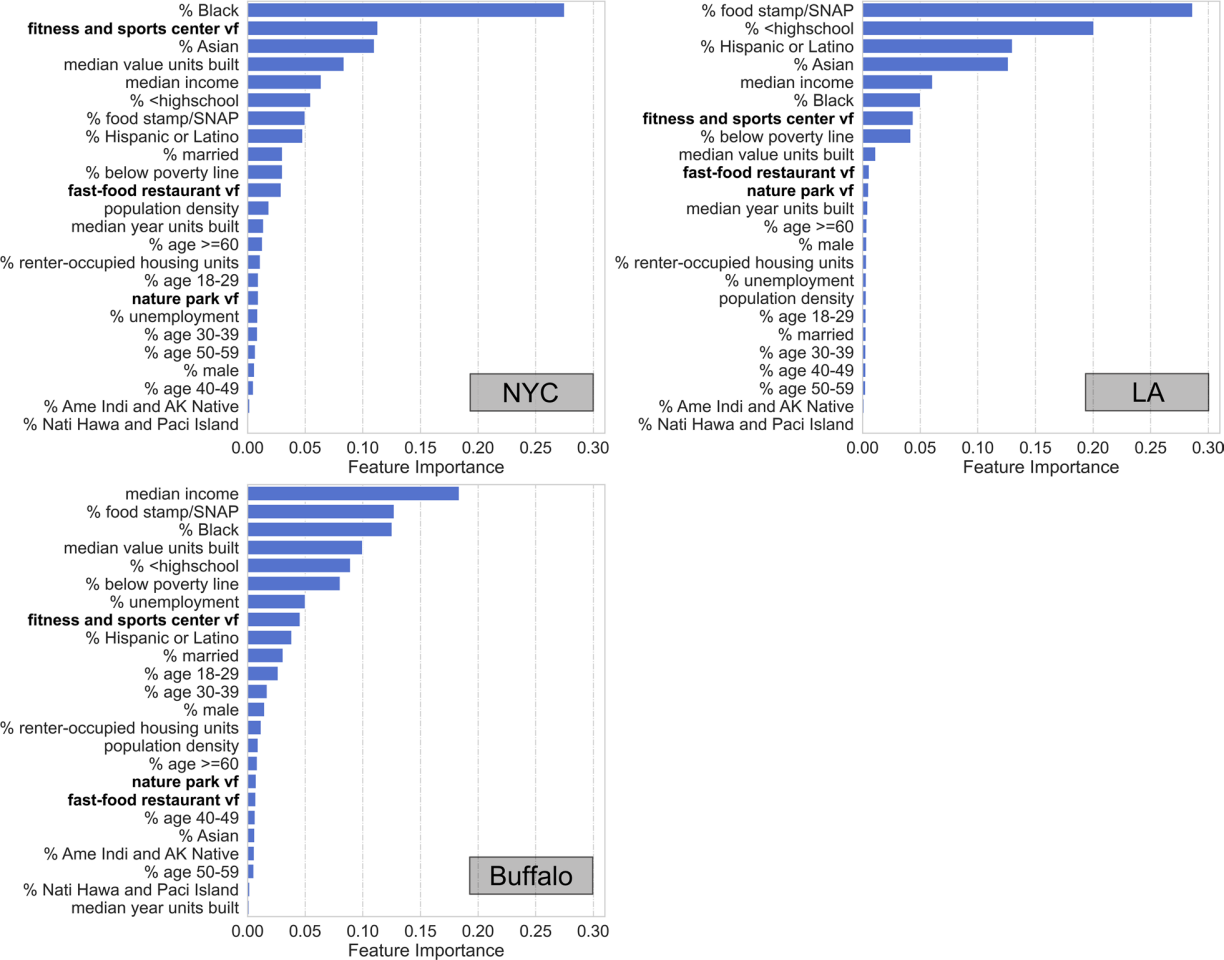
Feature importance of the independent variables output by the RF model for NYC, LA, and Buffalo

The RF model provides feature importance values indicating the relative importance of different input variables for helping the RF model predict neighborhood-level obesity prevalence. The importance values output by the model are normalized to the range of [0, 1] and sum up to 1. Figure 6 shows the feature importance values in the three cities. Since we have used tenfold cross-validation, 10 RF models are trained for each city which result in 10 sets of feature importance values. Figure 6 shows the mean importance value for each variable.

As can be seen in Fig. 6, the three diet and physical activity measurements derived from anonymized mobile phone location data play important roles in helping the RF model predict obesity prevalence, despite the fact that they bring only small improvements to the overall model performance as shown previously in Table 3. In particular, *fitness and sports center vf* is ranked as the 2nd most important feature in NYC, the 7th in LA, and the 8th in Buffalo among the 24 input variables. *Fast-food restaurant vf* is ranked as the 11th important variable in NYC, the 10th in LA, and the 18th in Buffalo. *Nature park vf* is ranked as the 17th in NYC, the 11th in LA, and the 17th in Buffalo. Note that unlike the regression coefficients obtained in the OLS model, feature importance from the RF model only tells us the relative importance of an input variable in helping the RF model make correct predictions, and does not indicate whether a variable is positively or negatively associated with obesity prevalence. The feature importance values for the socioeconomic and demographic variables are overall consistent with the results obtained from the OLS model. Variables related to poverty level (e.g., *median income, % food stamp/SNAP, and* % below poverty line), education (e.g., % < highschool), and racial and ethnic composition (e.g., *% Black* and *% Asian*) are all ranked as highly important features for the RF model to predict neighborhood-level obesity prevalence across the three cities.

In addition to OLS and RF, we have also obtained more detailed local regression coefficients and local feature importance from the GWR and GRF models respectively. However, due to the relatively small improvement brought by the derived three diet and physical activity measurements, we do not discuss them here to avoid making this paper longer and the results are included in Additional file 1: Figs. S2, S3. For the DNN model, it functions more like a “black box”, and we cannot directly obtain much information about the roles of individual input variables.

## Discussion

### Deriving neighborhood-level diet and physical activity measurements

Neighborhood-level diet and physical activity measurements, such as how neighborhood residents visit fast-food restaurants, fitness and sports centers, and nature parks, can be important information for supporting public health policies and decisions related to obesity prevention and intervention. Collecting such information typically requires considerable financial and labor resources, and even when such resources are available, the data collection process can take much time resulting in lags in the collected data (e.g., one or several years). In this study, we have proposed a method for deriving neighborhood-level diet and physical activity measurements from anonymized mobile phone location data. Because these data cover large geographic areas (e.g., the entire United States) and are being collected continuously with small temporal lags (e.g., within one or a few months), this method has potential to be applied to deriving diet and physical activity measurements for large geographic areas. We have demonstrated the feasibility of this method in three different US cities. The derived neighborhood-level measurements show consistent geographic patterns with the obesity prevalence from the CDC PLACES Project, i.e., neighborhoods with higher obesity prevalence tend to have higher fast-food restaurant visit frequencies and lower fitness center and nature park visit frequencies. To further quantify this consistency, we perform correlation analysis between the derived three types of diet and physical activity measurements and obesity prevalence. Considering that the relations may not be linear, we perform both Pearson’s and Spearman’s correlation, and the results are reported in Table 5. The correlation analysis results show that the three derived measurements are overall correlated with obesity prevalence in the three cities (although there is an exception in Buffalo). In particular, Fast-food restaurant visit frequency has a weak correlation with obesity prevalence in NYC and LA (with coefficients ranging between 0.283 and 0.331); fitness and sports center visit frequency has a strong correlation with obesity prevalence in all three cities (with coefficients ranging between – 0.803 and – 0.628); and nature park visit frequency has a weak to moderate correlation with obesity prevalence in all three cities (with coefficients ranging between – 0.514 and – 0.272). Results from the two types of correlation analyses are also consistent.

**Table 5:** Correlation coefficients between the three types of diet and physical activity measurements and obesity prevalence

	Pearson’s correlation			Spearman’s correlation		
	NYC	LA	Buffalo	NYC	LA	Buffalo
Fast-food restaurant vf	0.291***	0.283***	0.110	0.299***	0.331***	0.037
Fitness and sports center vf	− 0.628***	− 0.692***	− 0.651***	− 0.653***	− 0.803***	− 0.720***
Nature park vf	− 0.294***	− 0.460***	− 0.299**	− 0.328***	− 0.514***	− 0.272*

*p-value < 0.05; **p-value < 0.01; ***p-value < 0.001

### Implications for neighborhood-level obesity estimation

Accurately estimating neighborhood-level obesity prevalence has important meaning. Existing evidence has shown that neighborhood environments can directly or indirectly influence the health behaviors of neighborhood residents [10, 11, 14, 17, 18]. Knowing the neighborhoods that are more likely to suffer from high obesity prevalence therefore allows obesity prevention and intervention programs to focus on these neighborhoods and potentially improve their environments. This can be especially helpful when resources are limited, and effort focusing on a smaller number of neighborhoods could have a higher positive impact than effort more evenly distributed throughout an entire city.

The three neighborhood-level diet and physical activity measurements derived from anonymized mobile phone data in this study are correlated with neighborhood-level obesity prevalence, as shown in Table 5. However, they provide only small improvements to obesity estimation compared with using 21 socioeconomic and demographic variables. This result suggests that the information provided by the three derived measurements possibly overlap with the other socioeconomic and demographic variables. To further understand this possibility, we perform correlation analyses between the three derived diet and physical activity measures and the other socioeconomic and demographic variables, and the results are reported in Additional file 1: Tables S4–S6. In particular, *fast-food restaurant vf* shows a weak to moderate correlation with *median value units built* (with coefficients ranging from − 0.504 to − 0.312); *fitness and sports center vf* shows a moderate to strong correlation with *% food stamp/SNAP* (with coefficients ranging from − 0.798 to − 0.499), and a weak to strong correlation with *%* < *highschool* (with coefficients ranging from − 0.846 to − 0.323); and *nature park vf* shows a weak to moderate correlation with *median income* (with coefficients ranging from 0.231 to 0.597). Overall, the results show that the three derived measurements are correlated with many socioeconomic and demographic variables, especially those related to poverty level, education, and median housing value.

We also perform stepwise regression analysis to further understand the socioeconomic and demographic variables that may be redundant with the three derived measurements. We start with the three derived measurements and gradually add the other socioeconomic and demographic variables in a stepwise manner. The results are reported in Additional file 1: Tables S7–S9. Three highly interesting observations can be obtained. First, using the three derived diet and physical activity measurements alone can already provide a moderate estimation accuracy for obesity prevalence at the neighborhood level. When starting with the three derived measurements, we can already achieve an R^2^ of 0.437 for NYC, 0.486 for LA, and 0.480 for Buffalo. Second, *fast-food restaurant vf* and *fitness and sports center vf* play important roles for the models to estimate neighborhood-level obesity prevalence in NYC and LA. These two measurements were kept in all the steps and the final models, after being compared with other socioeconomic and demographic variables in the stepwise regression. Interestingly, *nature park vf* became insignificant and was dropped from the models after the addition of *% Black* in both NYC and LA. This result suggests that there might be a high level of redundancy between *% Black* and *nature park vf* in NYC and LA, which is worth future investigations. Third, the result of Buffalo seems to be quite different from the results of NYC and LA in that all three measurements were eventually dropped by the model. In particular, *nature park vf* and *fast-food restaurant vf* were dropped after the addition of *% food stamp/SNAP*, and *fitness and sports center vf* was dropped after the addition of *median value units built*. Both *% food stamp/SNAP* and *median value units built* are linked to poverty, and this result suggests that poverty may be more predictive of health behaviors in Buffalo than in NYC and LA where *fast-food restaurant vf* and *fitness and sports center vf* were kept in the final models along with *% food stamp/SNAP* and *median value units built*.

Given the small improvements brought by the derived diet and physical activity measurements, it seems less needed to include these measurements for obesity estimation when we already have socioeconomic and demographic data. Then, what other values could be brought by the derived diet and physical activity measurements? We think there are at least three other situations under which these derived measurements can be useful. First, when only limited socioeconomic and demographic data are available, these derived measurements may provide stronger enhancement for neighborhood-level obesity estimation. It is worth noting that the small improvements obtained in our results are based on 21 socioeconomic and demographic variables. While such comprehensive data are available in the United States, they are not always available in many other countries, especially developing countries. Meanwhile, mobile phone location data seems to be available in some developing countries [66, 67]. As shown in the stepwise regression analysis, using the three derived measurements alone already provides a moderate accuracy for neighborhood-level obesity estimation. When only a few socioeconomic and demographic variables are available (or no data is available at all), diet and physical activity measurements derived from mobile phone location data may help provide better enhancement for obesity estimation. Second, the derived measurements can be used as the dependent or outcome variables to study health behaviors. For example, they can be used in studies that aim to understand the factors that affect the visit frequency of neighborhood residents to fast-food restaurants, or in studies that aim to evaluate the extent of a prevention strategy, such as park renovation [68], in improving related health behaviors, such as increased park visits from nearby neighborhoods. Third, these diet and physical activity measurements, given their ability to link neighborhoods and related places (e.g., fast-food restaurants), can help identify the places that may be the primary contributors to obesity. For example, they can help answer the question: *which fast-food restaurants are mostly visited by the residents of a neighborhood with a high obesity prevalence*? The answer may not be the fast-food restaurant that has the shortest distance to the neighborhood. Identifying these primary contributing places can help investigate the underlying issues and use suitable prevention strategies at these places, e.g., requiring fast-food restaurants to make the caloric content of foods visible on menu boards if this was not done yet.

### Methodological implications

This study also sheds light on neighborhood-level obesity estimation from a methodological perspective. We tested five different models, including both statistical and machine learning models, across three different cities. We included machine learning models in addition to statistical models because there is an increasing interest in using AI for health studies and in particular for obesity estimation [51, 69–71]. Overall, the three machine learning models performed better than the OLS model but not as good as the GWR model. The outstanding performance of GWR can be attributed to its ability to explicitly model spatial autocorrelation, a local effect we have observed in neighborhood-level obesity prevalence during the analysis stage. While deep learning models have demonstrated outstanding performances in image recognition and natural language processing [64], their performance on tabular data (i.e., data structured into rows and columns, such as those used in this study) seems to be similar to statistical models and other “shallow learning” models such as random forest. Similar results have also been reported in the literature [72–74]. Among the five models, GWR is a spatial statistical model which performed better than the non-spatial OLS model, and GRF is a spatial machine learning model which performed better than the two other non-spatial machine learning models, i.e., RF and DNN. This result suggests that spatial models should be preferred when spatial autocorrelation exists in obesity prevalence data. In terms of the computing processes, fitting the two statistical models took less time compared with training the three machine learning models, likely due to their simpler model architectures. While this study shows that GWR is the best among the five tested models for neighborhood-level obesity estimation in both prediction accuracy and computing time, more research is needed to further test these models in other cities based on other datasets.

### Limitations

This study is not without limitations. First, we have used census tracts as the geographic units for analysis, because the obesity data from the CDC PLACES project are at this geographic level. While census tracts are overall sufficient for this current study, results at finer geographic units, such as census block groups, may allow us to identify neighborhoods that have obesity issues more accurately and to develop more precise prevention strategies. When new data have become available, future studies could be conducted at the census block group level. Second, this study has focused on three cities, namely NYC, LA, and Buffalo, which are located in different geographic areas and have different city sizes. We could extend this study to other cities to examine the roles of place visits related to diet and physical activity in enhancing obesity prevalence prediction. Given the larger difference between Buffalo and the other two megacities shown in the results of this study, it would be especially interesting to include more mid-sized or small cities in future research.

## Conclusions

This study investigates the feasibility of deriving neighborhood-level diet and physical activity measurements from anonymized mobile phone location data and their ability to enhance obesity estimation. We have proposed a method for deriving neighborhood-level diet and physical activity measurements by leveraging anonymized mobile phone location data, POI data, and census tracts. We have conducted case studies in three different US cities, namely NYC, LA, and Buffalo, using five different statistical and machine learning models. We find that it is feasible to derive neighborhood-level diet and physical activity measurements from anonymized mobile phone location data. These derived measurements provide only small enhancement for obesity estimation compared with using a comprehensive set of 21 neighborhood-level socioeconomic and demographic variables. However, the derived measurements are overall correlated with neighborhood-level obesity prevalence from the CDC PLACES project across the three cities. Also, using the three derived measurements alone can already provide a moderate accuracy for obesity estimation. These derived diet and physical activity measurements may provide a stronger enhancement when comprehensive socioeconomic and demographic data are not available (e.g., in some developing countries). They can also be used for studying health behaviors and identifying primary places contributing to obesity-related issues.

## Supplementary Information

**Additional file 1: Table S1.** The total numbers of POIs for each of the three types of places used to derive diet and physical activity measurements in NYC, LA, and Buffalo. **Figure S2.** Local regression coefficients of the three derived diet and physical activity measurements by the GWR model for NYC, LA, and Buffalo. **Figure S3.** Local feature importance of the three derived diet and physical activity measurements by the GRF model for NYC, LA, and Buffalo. **Table S4.** Correlation coefficients between the fast-food restaurant visit frequency and other independent variables in NYC, LA, and Buffalo. **Table S5. **Correlation coefficients between fitness and sports center visit frequency and other independent variables in NYC, LA, and Buffalo. **Table S6.** Correlation coefficients between nature park visit frequency and other independent variables in NYC, LA, and Buffalo. **Table S7.** Stepwise regression result of NYC. **Table S8. **Stepwise regression result of LA. **Table S9.** Stepwise regression result of Buffalo.

## Data Availability

Data related to the analysis results from this study are available from the authors upon request. The original anonymized mobile phone location data was provided by the company SafeGraph and interested readers may contact SafeGraph for data access. Obesity data at the census tract level from the CDC PLACES Project are publicly available at: https://chronicdata.cdc.gov/500-Cities-Places/PLACES-Census-Tract-Data-GIS-Friendly-Format-2021-/yjkw-uj5s. Socioeconomic and demographic data from the American Community Survey (ACS) of the US Census Bureau are publicly available at: https://data.census.gov/cedsci/.

## Abbreviations

POIs: Points of interest
NYC: New York City
LA: Los Angeles
CDC: Centers for Disease Control and Prevention
ACS: American Community Survey
SNAP: Supplemental nutrition assistance program
OLS: Ordinary least squares
GWR: Geographically weighted regression
RF: Random forest
DNN: Deep neural network
GRF: Geographical random forests
AI: Artificial Intelligence
RMSE: Root mean square error
AIC: Akaike information criterion
VIF: Variance inflation factor
