# Supplementary material for: Deriving neighborhood-level diet and physical activity measurements from anonymized mobile phone location data for enhancing obesity estimation
Source: Int J Health Geogr. 2022 Dec 30;21:22. doi: 10.1186/s12942-022-00321-4 (PMC9801358; doi:10.1186/s12942-022-00321-4)
Supplement: Supplementary file 1 — Additional file 1: Table S1. The total numbers of POIs for each of the three types of places used to derive diet and physical activity measurements in NYC, LA, and Buffalo. Figure S2. Local regression coefficients of the three derived diet and physical activity measurements by the GWR model for NYC, LA, and Buffalo. Figure S3. Local feature importance of the three derived diet and physical activity measurements by the GRF model for NYC, LA, and Buffalo. Table S4. Correlation coefficients between the fast-food restaurant visit frequency and other independent variables in NYC, LA, and Buffalo. Table S5. Correlation coefficients between fitness and sports center visit frequency and other independent variables in NYC, LA, and Buffalo. Table S6. Correlation coefficients between nature park visit frequency and other independent variables in NYC, LA, and Buffalo. Table S7. Stepwise regression result of NYC. Table S8. Stepwise regression result of LA. Table S9. Stepwise regression result of Buffalo. [file 12942_2022_321_MOESM1_ESM.docx]

**Additional file 1**

Table S1. The total numbers of POIs for each of the three types of places used to derive diet and physical activity measurements in NYC, LA, and Buffalo.

| **Place type** | **NYC** | **LA** | **Buffalo** |
| --- | --- | --- | --- |
| fast-food restaurant | 25355 | 16950 | 1884 |
| fitness and sports center | 21550 | 16443 | 1255 |
| nature park | 24857 | 17559 | 2153 |


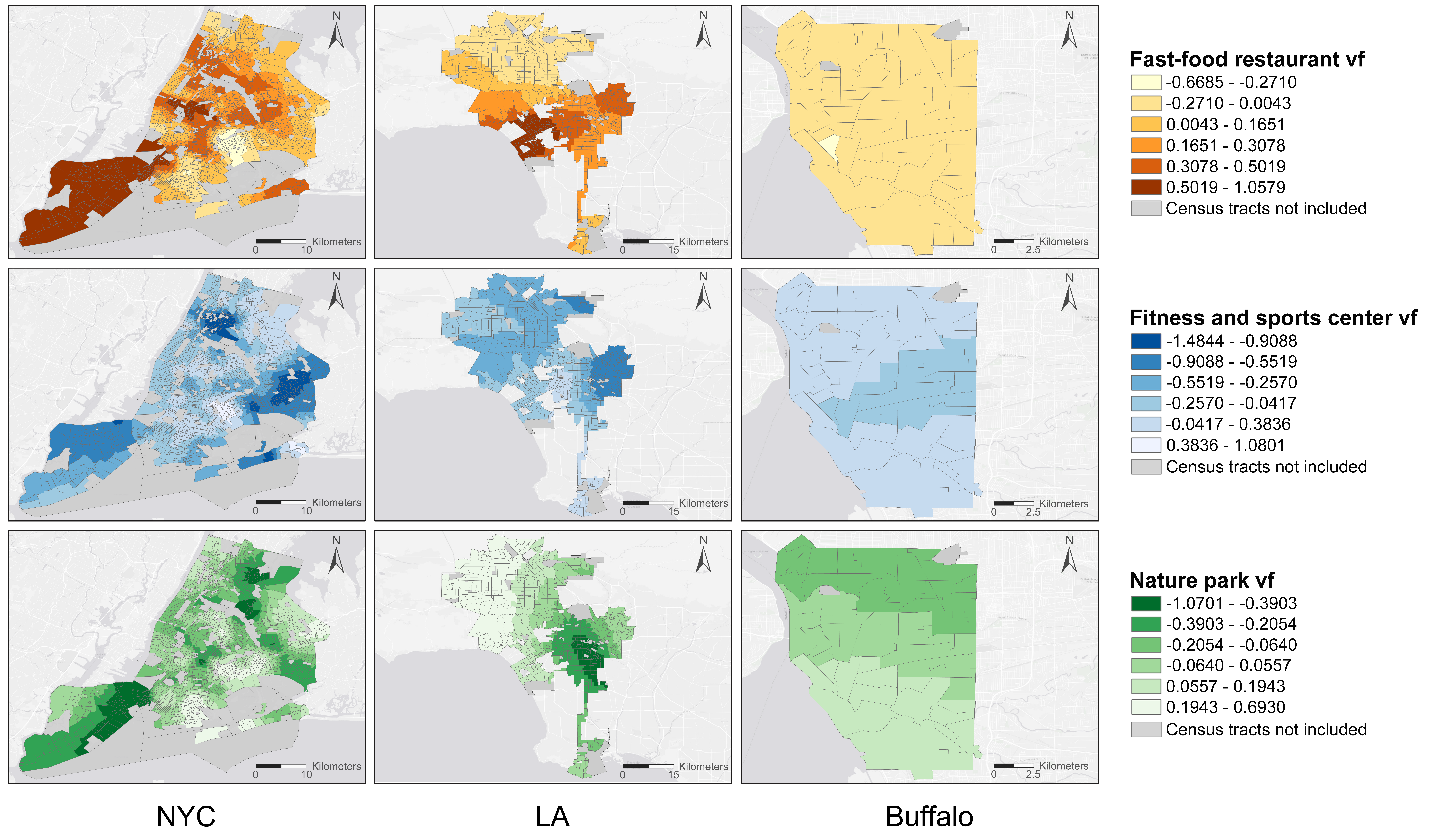
Figure S2. Local regression coefficients of the three derived diet and physical activity measurements by the GWR model for NYC, LA, and Buffalo.


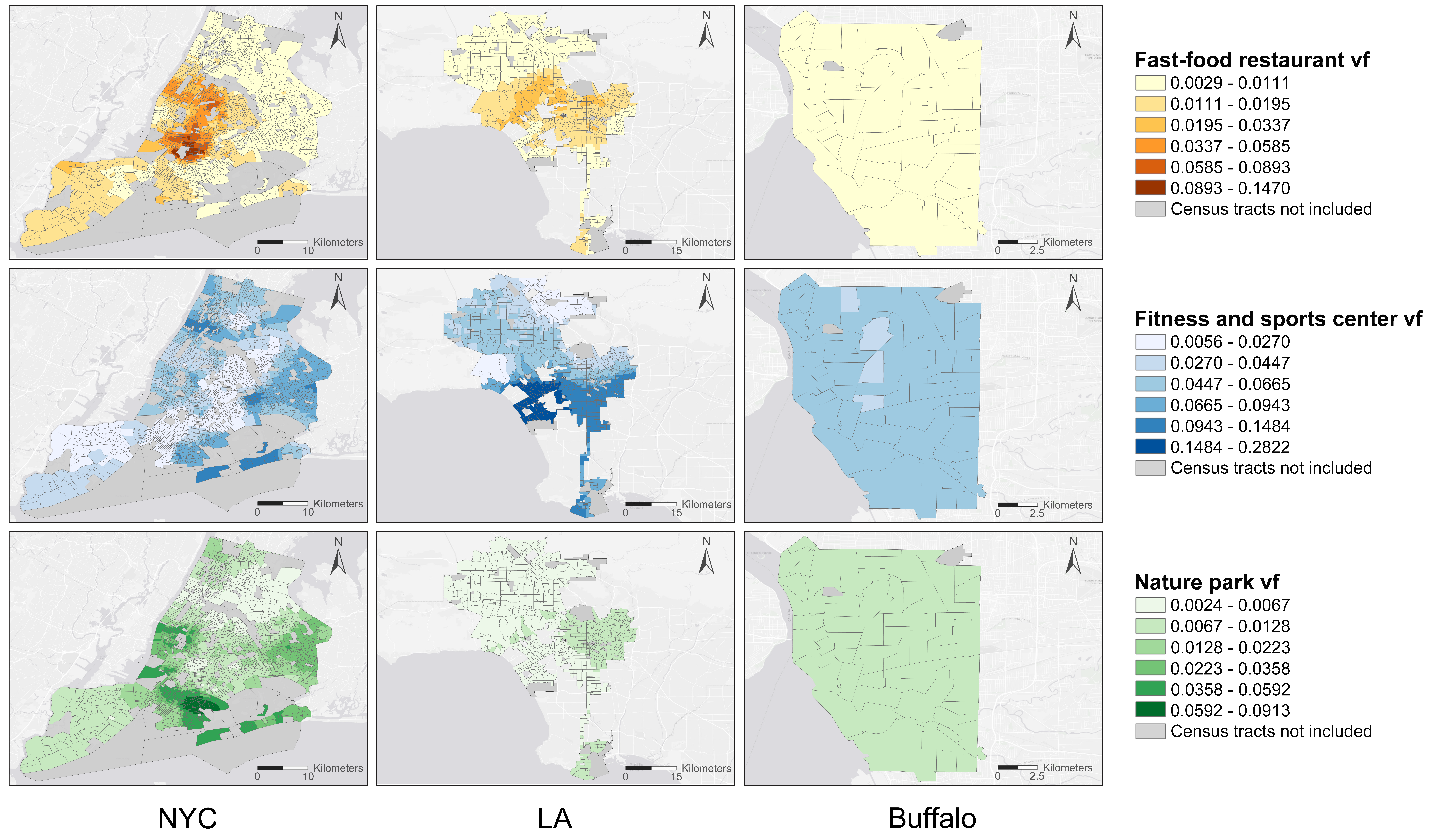


Figure S3. Local feature importance of the three derived diet and physical activity measurements by the GRF model for NYC, LA, and Buffalo.

Table S4. Correlation coefficients between the fast-food restaurant visit frequency and other independent variables in NYC, LA, and Buffalo.

|  | **Pearson’s correlation** | | | **Spearman’s correlation** | | |
| --- | --- | --- | --- | --- | --- | --- |
|  | **NYC** | **LA** | **Buffalo** | **NYC** | **LA** | **Buffalo** |
| % Black | 0.1042*** | 0.1049** | 0.1021 | 0.0741*** | 0.0820* | 0.0052 |
| % Ame Indi and AK Native | -0.0133 | -0.0204 | 0.0497 | 0.0062 | 0.0575 | -0.0768 |
| % Asian | 0.0499* | -0.0966** | -0.2028 | 0.0331 | -0.0919** | -0.0577 |
| % Nati Hawa and Paci Island | 0.0166 | 0.0031 | 0.0843 | 0.0217 | -0.0386 | 0.0205 |
| % Hispanic or Latino | 0.0956*** | 0.3295*** | -0.0399 | 0.1342*** | 0.3475*** | -0.0926 |
| % male | -0.0278 | -0.0058 | -0.193 | -0.0173 | -0.0246 | -0.1409 |
| % married | 0.1208*** | 0.1931*** | 0.0184 | 0.1143*** | 0.2267*** | 0.063 |
| % age 18-29 | -0.1503*** | -0.0750* | -0.1252 | -0.1226*** | -0.0658* | -0.1652 |
| % age 30-39 | -0.2970*** | -0.3553*** | -0.2322* | -0.2661*** | -0.3349*** | -0.1336 |
| % age 40-49 | 0.1065*** | -0.0757* | -0.0414 | 0.1186*** | -0.1010** | -0.0212 |
| % age 50-59 | 0.2967*** | 0.1994*** | 0.1129 | 0.3051*** | 0.1968*** | 0.1179 |
| % age >=60 | 0.0922*** | -0.0509 | 0.0431 | 0.1413*** | -0.0287 | 0.0807 |
| % <highschool | 0.0686** | 0.2416*** | -0.0086 | 0.1259*** | 0.3061*** | -0.0086 |
| med income | -0.1705*** | -0.1913*** | -0.1551 | -0.0516* | -0.1154*** | -0.0824 |
| % unemployment | 0.0870*** | 0.0460 | 0.1443 | 0.1185*** | 0.0640* | 0.1605 |
| % below poverty line | -0.1266*** | 0.0224 | 0.0178 | -0.1056*** | 0.0629 | -0.0312 |
| % food stamp/SNAP | -0.0291 | 0.2097*** | 0.1045 | 0.0209 | 0.2713*** | 0.0314 |
| median value units built | -0.4059*** | -0.5044*** | -0.3123** | -0.3545*** | -0.4396*** | -0.1705 |
| median year units built | 0.1150*** | 0.0902** | 0.0167 | 0.1556*** | 0.0914** | 0.1632 |
| % renter-occupied housing units | -0.2965*** | -0.2457*** | -0.2027 | -0.2713*** | -0.2523*** | -0.2353* |
| population density | -0.2692*** | -0.0976** | -0.0342 | -0.2958*** | -0.0815* | -0.0292 |
| *p-value < 0.05; **p-value < 0.01; ***p-value < 0.001 | | | | | | |

Table S5. Correlation coefficients between fitness and sports center visit frequency and other independent variables in NYC, LA, and Buffalo.

|  | **Pearson’s correlation** | | | **Spearman’s correlation** | | |
| --- | --- | --- | --- | --- | --- | --- |
|  | **NYC** | **LA** | **Buffalo** | **NYC** | **LA** | **Buffalo** |
| % Black | -0.4389*** | -0.2139*** | -0.5320*** | -0.5231*** | -0.1240*** | -0.5794*** |
| % Ame Indi and AK Native | -0.0777*** | -0.1295*** | 0.0624 | -0.0996*** | -0.1937*** | 0.0365 |
| % Asian | 0.0378 | 0.1526*** | 0.0292 | 0.2941*** | 0.4313*** | 0.0665 |
| % Nati Hawa and Paci Island | -0.0073 | -0.0207 | 0.0133 | 0.0215 | 0.0170 | 0.0374 |
| % Hispanic or Latino | -0.3075*** | -0.7529*** | 0.0293 | -0.2652*** | -0.8298*** | 0.1543 |
| % male | 0.0296 | -0.0629 | 0.2286* | 0.0879*** | -0.0237 | 0.2286* |
| % married | 0.1512*** | 0.2787*** | 0.2947** | 0.1922*** | 0.2180*** | 0.2957** |
| % age 18-29 | -0.0223 | -0.1194*** | 0.2488* | -0.1162*** | -0.2432*** | 0.3408** |
| % age 30-39 | 0.3095*** | 0.0914** | 0.2701* | 0.2990*** | 0.1815*** | 0.2857* |
| % age 40-49 | 0.0980*** | 0.1014** | 0.0168 | 0.1058*** | 0.1505*** | 0.0303 |
| % age 50-59 | -0.1018*** | 0.1333*** | -0.1093 | -0.0939*** | 0.1761*** | -0.1070 |
| % age >=60 | 0.2027*** | 0.4389*** | -0.0217 | 0.1831*** | 0.4602*** | 0.0057 |
| % <highschool | -0.5188*** | -0.7226*** | -0.3232** | -0.5321*** | -0.8463*** | -0.4377*** |
| med income | 0.6362*** | 0.6931*** | 0.5615*** | 0.5141*** | 0.6593*** | 0.6128*** |
| % unemployment | -0.3688*** | -0.2344*** | -0.5185*** | -0.3964*** | -0.2647*** | -0.5813*** |
| % below poverty line | -0.3985*** | -0.4863*** | -0.4776*** | -0.4080*** | -0.6225*** | -0.5051*** |
| % food stamp/SNAP | -0.4993*** | -0.6534*** | -0.5307*** | -0.5685*** | -0.7976*** | -0.5656*** |
| median value units built | 0.5571*** | 0.7463*** | 0.4816*** | 0.4693*** | 0.7624*** | 0.6876*** |
| median year units built | 0.1010*** | 0.2818*** | -0.0749 | 0.0245 | 0.3431*** | 0.0507 |
| % renter-occupied housing units | -0.0713** | -0.2553*** | -0.1846 | -0.0800*** | -0.1805*** | -0.1551 |
| population density | 0.1604*** | -0.2075*** | 0.2846* | 0.0600** | -0.3091*** | 0.3137** |
| *p-value < 0.05; **p-value < 0.01; ***p-value < 0.001 | | | | | | |

Table S6. Correlation coefficients between nature park visit frequency and other independent variables in NYC, LA, and Buffalo.

|  | **Pearson’s correlation** | | | **Spearman’s correlation** | | |
| --- | --- | --- | --- | --- | --- | --- |
|  | **NYC** | **LA** | **Buffalo** | **NYC** | **LA** | **Buffalo** |
| % Black | -0.3400*** | -0.3133*** | -0.2271* | -0.3581*** | -0.3381*** | -0.2861* |
| % Ame Indi and AK Native | -0.0496* | -0.0135 | -0.0144 | -0.0510* | -0.0278 | 0.0237 |
| % Asian | -0.0304 | 0.0657* | -0.2255* | 0.1094*** | 0.2287*** | -0.1946 |
| % Nati Hawa and Paci Island | 0.0151 | -0.0281 | 0.0855 | 0.0398 | -0.0214 | 0.0396 |
| % Hispanic or Latino | -0.1711*** | -0.401*** | -0.0058 | -0.1230*** | -0.4193*** | 0.0622 |
| % male | 0.0330 | -0.0570 | 0.0600 | 0.0343 | -0.0496 | 0.0274 |
| % married | 0.2392*** | 0.4117*** | 0.1874 | 0.2252*** | 0.3530*** | 0.1187 |
| % age 18-29 | -0.1613*** | -0.2795*** | 0.0612 | -0.2402*** | -0.3386*** | -0.0502 |
| % age 30-39 | -0.0108 | -0.0794* | 0.0170 | 0.0103 | -0.0307 | 0.0222 |
| % age 40-49 | 0.0632** | 0.0804* | -0.1318 | 0.0636** | 0.0778* | -0.1389 |
| % age 50-59 | 0.0991*** | 0.2003*** | 0.1066 | 0.0599** | 0.1965*** | 0.1438 |
| % age >=60 | 0.2473*** | 0.4150*** | 0.1005 | 0.2341*** | 0.3988*** | 0.1128 |
| % <highschool | -0.3420*** | -0.4317*** | -0.2795* | -0.3438*** | -0.4834*** | -0.2362* |
| med income | 0.3692*** | 0.5974*** | 0.2305* | 0.3109*** | 0.5536*** | 0.1814 |
| % unemployment | -0.1770*** | -0.1503*** | -0.0926 | -0.1879*** | -0.1448*** | -0.0138 |
| % below poverty line | -0.2490*** | -0.4016*** | -0.2486* | -0.2475*** | -0.4960*** | -0.2044 |
| % food stamp/SNAP | -0.2792*** | -0.4241*** | -0.2499* | -0.3235*** | -0.5100*** | -0.2272* |
| median value units built | 0.1636*** | 0.5194*** | 0.2695* | 0.1134*** | 0.4778*** | 0.1805 |
| median year units built | 0.2346*** | 0.1386*** | 0.1013 | 0.1956*** | 0.1802*** | -0.0335 |
| % renter-occupied housing units | -0.2251*** | -0.3892*** | -0.1951 | -0.1581*** | -0.3685*** | -0.1663 |
| population density | -0.1266*** | -0.3302*** | -0.0602 | -0.1531*** | -0.4229*** | -0.0426 |
| *p-value < 0.05; **p-value < 0.01; ***p-value < 0.001 | | | | | | |

Table S7. Stepwise regression result of NYC.

|  | **------Step 1-----** | | | | | | **------Step 2-----** | | | | | | **------Step 3-----** | | | | | |
| --- | --- | --- | --- | --- | --- | --- | --- | --- | --- | --- | --- | --- | --- | --- | --- | --- | --- | --- |
|  | **Coef** | **P** | | | | | **Coef** | | | **P** | | | **Coef** | | | **P** | | |
| Constant | 25.6815 |  | | | | | 25.6815 | | |  | | | 25.6815 | | |  | | |
| fast-food restaurant vf | 1.265 | 0.000 | | | | | 1.4935 | | | 0.000 | | | 1.6628 | | | 0.000 | | |
| fitness and sports center vf | -3.400 | 0.000 | | | | | -3.1245 | | | 0.000 | | | -2.1780 | | | 0.000 | | |
| nature park vf | -0.227 | 0.059 | | | | | -0.4936 | | | 0.000 | | | -0.4612 | | | 0.000 | | |
| % Asian |  |  | | | | | -2.8341 | | | 0.000 | | | -2.4932 | | | 0.000 | | |
| % food stamp/SNAP |  |  | | | | |  | | |  | | | 1.9057 | | | 0.000 | | |
| % Black |  |  | | | | |  | | |  | | |  | | |  | | |
| % Hispanic or Latino |  |  | | | | |  | | |  | | |  | | |  | | |
| % below poverty line |  |  | | | | |  | | |  | | |  | | |  | | |
| % renter-occupied housing units |  |  | | | | |  | | |  | | |  | | |  | | |
| median income |  |  | | | | |  | | |  | | |  | | |  | | |
| % age >=60 |  |  | | | | |  | | |  | | |  | | |  | | |
| % age 18-29 |  |  | | | | |  | | |  | | |  | | |  | | |
| population density |  |  | | | | |  | | |  | | |  | | |  | | |
| median year units built |  |  | | | | |  | | |  | | |  | | |  | | |
| % <highschool |  |  | | | | |  | | |  | | |  | | |  | | |
| median value units built |  |  | | | | |  | | |  | | |  | | |  | | |
| % age 30-39 |  |  | | | | |  | | |  | | |  | | |  | | |
| % age 50-59 |  |  | | | | |  | | |  | | |  | | |  | | |
| % age 40-49 |  |  | | | | |  | | |  | | |  | | |  | | |
|  |  |  | | | | |  | | |  | | |  | | |  | | |
| S |  | 4.42160 | | | | |  | | | 3.40547 | | |  | | | 3.00385 | | |
| R-sq |  | 43.67% | | | | |  | | | 66.60% | | |  | | | 74.03% | | |
| R-sq(adj) |  | 43.59% | | | | |  | | | 66.54% | | |  | | | 73.96% | | |
| Mallows’ Cp |  | 6508.39 | | | | |  | | | 3051.88 | | |  | | | 1933.92 | | |
| AICc |  | 11598.73 | | | | |  | | | 10557.88 | | |  | | | 10058.19 | | |
| BIC |  | 11626.69 | | | | |  | | | 10591.43 | | |  | | | 10097.32 | | |
|  | **-----Step 4-----** | | | | **-----Step 5-----** | | | | | | **-----Step 6-----** | | | | | |  |  |
|  | **Coef** | | **P** | | **Coef** | | | **P** | | | **Coef** | | | **P** | | |  |  |
| Constant | 25.6815 | |  | | 25.6815 | | |  | | | 25.6815 | | |  | | |  |  |
| fast-food restaurant vf | 1.4424 | | 0.000 | | 1.4310 | | | 0.000 | | | 1.2741 | | | 0.000 | | |  |  |
| fitness and sports center vf | -1.6140 | | 0.000 | | -1.6319 | | | 0.000 | | | -1.3443 | | | 0.000 | | |  |  |
| nature park vf | -0.0419 | | 0.577 | |  | | |  | | |  | | |  | | |  |  |
| % Asian | -1.6555 | | 0.000 | | -1.6479 | | | 0.000 | | | -1.3229 | | | 0.000 | | |  |  |
| % food stamp/SNAP | 1.9981 | | 0.000 | | 1.9993 | | | 0.000 | | | 1.4582 | | | 0.000 | | |  |  |
| % Black | 1.8089 | | 0.000 | | 1.8196 | | | 0.000 | | | 2.3733 | | | 0.000 | | |  |  |
| % Hispanic or Latino |  | |  | |  | | |  | | | 1.1372 | | | 0.000 | | |  |  |
| % below poverty line |  | |  | |  | | |  | | |  | | |  | | |  |  |
| % renter-occupied housing units |  | |  | |  | | |  | | |  | | |  | | |  |  |
| median income |  | |  | |  | | |  | | |  | | |  | | |  |  |
| % age >=60 |  | |  | |  | | |  | | |  | | |  | | |  |  |
| % age 18-29 |  | |  | |  | | |  | | |  | | |  | | |  |  |
| population density |  | |  | |  | | |  | | |  | | |  | | |  |  |
| median year units built |  | |  | |  | | |  | | |  | | |  | | |  |  |
| % <highschool |  | |  | |  | | |  | | |  | | |  | | |  |  |
| median value units built |  | |  | |  | | |  | | |  | | |  | | |  |  |
| % age 30-39 |  | |  | |  | | |  | | |  | | |  | | |  |  |
| % age 50-59 |  | |  | |  | | |  | | |  | | |  | | |  |  |
| % age 40-49 |  | |  | |  | | |  | | |  | | |  | | |  |  |
|  |  | |  | |  | | |  | | |  | | |  | | |  |  |
| S |  | | 2.67169 | |  | | | 2.67123 | | |  | | | 2.53314 | | |  |  |
| R-sq |  | | 79.47% | |  | | | 79.46% | | |  | | | 81.54% | | |  |  |
| R-sq(adj) |  | | 79.40% | |  | | | 79.41% | | |  | | | 81.48% | | |  |  |
| Mallows’ Cp |  | | 1116.00 | |  | | | 1114.49 | | |  | | | 803.13 | | |  |  |
| AICc |  | | 9591.64 | |  | | | 9589.94 | | |  | | | 9379.17 | | |  |  |
| BIC |  | | 9636.35 | |  | | | 9629.07 | | |  | | | 9423.88 | | |  |  |
|  | **-----Step 7-----** | | | | **-----Step 8-----** | | | | | | **-----Step 9-----** | | | | | |  |  |
|  | **Coef** | | **P** | | **Coef** | | | **P** | | | **Coef** | | | **P** | | |  |  |
| Constant | 25.6815 | |  | | 25.6815 | | |  | | | 25.6815 | | |  | | |  |  |
| fast-food restaurant vf | 1.3990 | | 0.000 | | 1.1402 | | | 0.000 | | | 0.9727 | | | 0.000 | | |  |  |
| fitness and sports center vf | -1.3150 | | 0.000 | | -1.0045 | | | 0.000 | | | -0.7180 | | | 0.000 | | |  |  |
| nature park vf |  | |  | |  | | |  | | |  | | |  | | |  |  |
| % Asian | -1.3585 | | 0.000 | | -1.2580 | | | 0.000 | | | -1.3381 | | | 0.000 | | |  |  |
| % food stamp/SNAP | 0.467 | | 0.000 | | 0.672 | | | 0.000 | | | 0.521 | | | 0.000 | | |  |  |
| % Black | 2.4550 | | 0.000 | | 2.6016 | | | 0.000 | | | 2.5844 | | | 0.000 | | |  |  |
| % Hispanic or Latino | 1.0994 | | 0.000 | | 1.4327 | | | 0.000 | | | 1.4218 | | | 0.000 | | |  |  |
| % below poverty line | 1.206 | | 0.000 | | 1.598 | | | 0.000 | | | 1.367 | | | 0.000 | | |  |  |
| % renter-occupied housing units |  | |  | | -1.0355 | | | 0.000 | | | -1.1994 | | | 0.000 | | |  |  |
| median income |  | |  | |  | | |  | | | -0.7826 | | | 0.000 | | |  |  |
| % age >=60 |  | |  | |  | | |  | | |  | | |  | | |  |  |
| % age 18-29 |  | |  | |  | | |  | | |  | | |  | | |  |  |
| population density |  | |  | |  | | |  | | |  | | |  | | |  |  |
| median year units built |  | |  | |  | | |  | | |  | | |  | | |  |  |
| % <highschool |  | |  | |  | | |  | | |  | | |  | | |  |  |
| median value units built |  | |  | |  | | |  | | |  | | |  | | |  |  |
| % age 30-39 |  | |  | |  | | |  | | |  | | |  | | |  |  |
| % age 50-59 |  | |  | |  | | |  | | |  | | |  | | |  |  |
| % age 40-49 |  | |  | |  | | |  | | |  | | |  | | |  |  |
|  |  | |  | |  | | |  | | |  | | |  | | |  |  |
| S |  | | 2.44810 | |  | | | 2.34367 | | |  | | | 2.30760 | | |  |  |
| R-sq |  | | 82.77% | |  | | | 84.21% | | |  | | | 84.70% | | |  |  |
| R-sq(adj) |  | | 82.71% | |  | | | 84.15% | | |  | | | 84.63% | | |  |  |
| Mallows’ Cp |  | | 620.03 | |  | | | 403.82 | | |  | | | 331.94 | | |  |  |
| AICc |  | | 9243.94 | |  | | | 9071.01 | | |  | | | 9010.15 | | |  |  |
| BIC |  | | 9294.24 | |  | | | 9126.88 | | |  | | | 9071.60 | | |  |  |
|  | **-----Step 10----** | | | | | **-----Step 11----** | | | | | | **-----Step 12----** | | | | | |  |
|  | **Coef** | | | **P** | | **Coef** | | | **P** | | | **Coef** | | | **P** | | |  |
| Constant | 25.6815 | | |  | | 25.6815 | | |  | | | 25.6815 | | |  | | |  |
| fast-food restaurant vf | 0.9225 | | | 0.000 | | 0.8981 | | | 0.000 | | | 0.8309 | | | 0.000 | | |  |
| fitness and sports center vf | -0.5468 | | | 0.000 | | -0.5119 | | | 0.000 | | | -0.3499 | | | 0.000 | | |  |
| nature park vf |  | | |  | |  | | |  | | |  | | |  | | |  |
| % Asian | -1.3456 | | | 0.000 | | -1.3335 | | | 0.000 | | | -1.2962 | | | 0.000 | | |  |
| % food stamp/SNAP | 0.668 | | | 0.000 | | 0.531 | | | 0.000 | | | 0.592 | | | 0.000 | | |  |
| % Black | 2.5256 | | | 0.000 | | 2.5981 | | | 0.000 | | | 2.6684 | | | 0.000 | | |  |
| % Hispanic or Latino | 1.3081 | | | 0.000 | | 1.3231 | | | 0.000 | | | 1.4026 | | | 0.000 | | |  |
| % below poverty line | 1.201 | | | 0.000 | | 1.256 | | | 0.000 | | | 1.233 | | | 0.000 | | |  |
| % renter-occupied housing units | -1.4827 | | | 0.000 | | -1.3286 | | | 0.000 | | | -1.1304 | | | 0.000 | | |  |
| median income | -1.013 | | | 0.000 | | -1.0180 | | | 0.000 | | | -1.0311 | | | 0.000 | | |  |
| % age >=60 | -0.5180 | | | 0.000 | | -0.7566 | | | 0.000 | | | -0.7475 | | | 0.000 | | |  |
| % age 18-29 |  | | |  | | -0.5965 | | | 0.000 | | | -0.5991 | | | 0.000 | | |  |
| population density |  | | |  | |  | | |  | | | -0.4753 | | | 0.000 | | |  |
| median year units built |  | | |  | |  | | |  | | |  | | |  | | |  |
| % <highschool |  | | |  | |  | | |  | | |  | | |  | | |  |
| median value units built |  | | |  | |  | | |  | | |  | | |  | | |  |
| % age 30-39 |  | | |  | |  | | |  | | |  | | |  | | |  |
| % age 50-59 |  | | |  | |  | | |  | | |  | | |  | | |  |
| % age 40-49 |  | | |  | |  | | |  | | |  | | |  | | |  |
|  |  | | |  | |  | | |  | | |  | | |  | | |  |
| S |  | | | 2.27046 | |  | | | 2.21635 | | |  | | | 2.18452 | | |  |
| R-sq |  | | | 85.20% | |  | | | 85.90% | | |  | | | 86.31% | | |  |
| R-sq(adj) |  | | | 85.12% | |  | | | 85.83% | | |  | | | 86.23% | | |  |
| Mallows’ Cp |  | | | 259.15 | |  | | | 154.96 | | |  | | | 95.28 | | |  |
| AICc |  | | | 8946.42 | |  | | | 8851.21 | | |  | | | 8794.50 | | |  |
| BIC |  | | | 9013.45 | |  | | | 8923.80 | | |  | | | 8872.66 | | |  |
|  | **-----Step 13----** | | | | | **-----Step 14----** | | | | | | **-----Step 15----** | | | | | |  |
|  | **Coef** | | | **P** | | **Coef** | | | **P** | | | **Coef** | | | **P** | | |  |
| Constant | 25.6815 | | |  | | 25.6815 | | |  | | | 25.6815 | | |  | | |  |
| fast-food restaurant vf | 0.7874 | | | 0.000 | | 0.7876 | | | 0.000 | | | 0.7316 | | | 0.000 | | |  |
| fitness and sports center vf | -0.3857 | | | 0.000 | | -0.3462 | | | 0.000 | | | -0.2450 | | | 0.004 | | |  |
| nature park vf |  | | |  | |  | | |  | | |  | | |  | | |  |
| % Asian | -1.3075 | | | 0.000 | | -1.4498 | | | 0.000 | | | -1.4555 | | | 0.000 | | |  |
| % food stamp/SNAP | 0.588 | | | 0.000 | | 0.445 | | | 0.000 | | | 0.449 | | | 0.000 | | |  |
| % Black | 2.6475 | | | 0.000 | | 2.6298 | | | 0.000 | | | 2.6035 | | | 0.000 | | |  |
| % Hispanic or Latino | 1.4044 | | | 0.000 | | 1.2551 | | | 0.000 | | | 1.1531 | | | 0.000 | | |  |
| % below poverty line | 1.172 | | | 0.000 | | 1.108 | | | 0.000 | | | 1.153 | | | 0.000 | | |  |
| % renter-occupied housing units | -1.1631 | | | 0.000 | | -1.1259 | | | 0.000 | | | -1.0420 | | | 0.000 | | |  |
| median income | -1.0924 | | | 0.000 | | -1.0598 | | | 0.000 | | | -0.873 | | | 0.000 | | |  |
| % age >=60 | -0.7577 | | | 0.000 | | -0.7442 | | | 0.000 | | | -0.7673 | | | 0.000 | | |  |
| % age 18-29 | -0.5930 | | | 0.000 | | -0.5823 | | | 0.000 | | | -0.5937 | | | 0.000 | | |  |
| population density | -0.4220 | | | 0.000 | | -0.4352 | | | 0.000 | | | -0.4626 | | | 0.000 | | |  |
| median year units built | 0.2537 | | | 0.000 | | 0.2770 | | | 0.000 | | | 0.2289 | | | 0.000 | | |  |
| % <highschool |  | | |  | | 0.3958 | | | 0.000 | | | 0.4741 | | | 0.000 | | |  |
| median value units built |  | | |  | |  | | |  | | | -0.3423 | | | 0.000 | | |  |
| % age 30-39 |  | | |  | |  | | |  | | |  | | |  | | |  |
| % age 50-59 |  | | |  | |  | | |  | | |  | | |  | | |  |
| % age 40-49 |  | | |  | |  | | |  | | |  | | |  | | |  |
|  |  | | |  | |  | | |  | | |  | | |  | | |  |
| S |  | | | 2.17145 | |  | | | 2.16304 | | |  | | | 2.15164 | | |  |
| R-sq |  | | | 86.48% | |  | | | 86.59% | | |  | | | 86.74% | | |  |
| R-sq(adj) |  | | | 86.39% | |  | | | 86.50% | | |  | | | 86.64% | | |  |
| Mallows’ Cp |  | | | 71.63 | |  | | | 56.84 | | |  | | | 36.58 | | |  |
| AICc |  | | | 8771.58 | |  | | | 8757.12 | | |  | | | 8737.07 | | |  |
| BIC |  | | | 8855.32 | |  | | | 8846.42 | | |  | | | 8831.93 | | |  |
|  | **-----Step 16----** | | | | | **-----Step 17----** | | | | | | **-----Step 18----** | | | | | |  |
|  | **Coef** | | | **P** | | **Coef** | | | **P** | | | **Coef** | | | **P** | | |  |
| Constant | 25.6815 | | |  | | 25.6815 | | |  | | | 25.6815 | | |  | | |  |
| fast-food restaurant vf | 0.6996 | | | 0.000 | | 0.6893 | | | 0.000 | | | 0.6911 | | | 0.000 | | |  |
| fitness and sports center vf | -0.2280 | | | 0.007 | | -0.2418 | | | 0.004 | | | -0.2676 | | | 0.002 | | |  |
| nature park vf |  | | |  | |  | | |  | | |  | | |  | | |  |
| % Asian | -1.4421 | | | 0.000 | | -1.4862 | | | 0.000 | | | -1.5136 | | | 0.000 | | |  |
| % food stamp/SNAP | 0.378 | | | 0.001 | | 0.386 | | | 0.000 | | | 0.418 | | | 0.000 | | |  |
| % Black | 2.5961 | | | 0.000 | | 2.5526 | | | 0.000 | | | 2.5245 | | | 0.000 | | |  |
| % Hispanic or Latino | 1.1580 | | | 0.000 | | 1.1344 | | | 0.000 | | | 1.1081 | | | 0.000 | | |  |
| % below poverty line | 1.100 | | | 0.000 | | 1.122 | | | 0.000 | | | 1.151 | | | 0.000 | | |  |
| % renter-occupied housing units | -0.8564 | | | 0.000 | | -0.8558 | | | 0.000 | | | -0.888 | | | 0.000 | | |  |
| median income | -0.803 | | | 0.000 | | -0.815 | | | 0.000 | | | -0.823 | | | 0.000 | | |  |
| % age >=60 | -0.8874 | | | 0.000 | | -0.8725 | | | 0.000 | | | -0.8188 | | | 0.000 | | |  |
| % age 18-29 | -0.6513 | | | 0.000 | | -0.6094 | | | 0.000 | | | -0.5517 | | | 0.000 | | |  |
| population density | -0.4694 | | | 0.000 | | -0.4608 | | | 0.000 | | | -0.4517 | | | 0.000 | | |  |
| median year units built | 0.2430 | | | 0.000 | | 0.2538 | | | 0.000 | | | 0.2551 | | | 0.000 | | |  |
| % <highschool | 0.4545 | | | 0.000 | | 0.4640 | | | 0.000 | | | 0.4630 | | | 0.000 | | |  |
| median value units built | -0.3594 | | | 0.000 | | -0.3470 | | | 0.000 | | | -0.3369 | | | 0.000 | | |  |
| % age 30-39 | -0.2886 | | | 0.000 | | -0.2355 | | | 0.002 | | | -0.1932 | | | 0.017 | | |  |
| % age 50-59 |  | | |  | | 0.1507 | | | 0.012 | | | 0.1756 | | | 0.005 | | |  |
| % age 40-49 |  | | |  | |  | | |  | | | 0.0971 | | | 0.112 | | |  |
|  |  | | |  | |  | | |  | | |  | | |  | | |  |
| S |  | | | 2.14384 | |  | | | 2.14098 | | |  | | | 2.14015 | | |  |
| R-sq |  | | | 86.84% | |  | | | 86.89% | | |  | | | 86.90% | | |  |
| R-sq(adj) |  | | | 86.74% | |  | | | 86.77% | | |  | | | 86.78% | | |  |
| Mallows’ Cp |  | | | 23.11 | |  | | | 18.82 | | |  | | | 18.29 | | |  |
| AICc |  | | | 8723.61 | |  | | | 8719.32 | | |  | | | 8718.80 | | |  |
| BIC |  | | | 8824.03 | |  | | | 8825.30 | | |  | | | 8830.34 | | |  |
| α to enter = 0.15, α to remove = 0.15 | | | | | | | | | | | | | | | | | |  |

Table S8. Stepwise regression result of LA.

|  | **-----Step 1-----** | | | | **-----Step 2-----** | | | | **-----Step 3-----** | | | |  |
| --- | --- | --- | --- | --- | --- | --- | --- | --- | --- | --- | --- | --- | --- |
|  | **Coef** | **P** | | | **Coef** | | **P** | | **Coef** | | **P** | |  |
| Constant | 27.429 |  | | | 27.4290 | |  | | 27.4290 | |  | |  |
| fast-food restaurant vf | 0.398 | 0.004 | | | 0.4010 | | 0.000 | | 0.3124 | | 0.000 | |  |
| fitness and sports center vf | -3.340 | 0.000 | | | -0.973 | | 0.000 | | -1.212 | | 0.000 | |  |
| nature park vf | -0.482 | 0.003 | | | -0.282 | | 0.010 | | -0.3818 | | 0.000 | |  |
| % food stamp/SNAP |  |  | | | 3.805 | | 0.000 | | 2.9545 | | 0.000 | |  |
| % Asian |  |  | | |  | |  | | -1.8794 | | 0.000 | |  |
| % below poverty line |  |  | | |  | |  | |  | |  | |  |
| % Black |  |  | | |  | |  | |  | |  | |  |
| % Hispanic or Latino |  |  | | |  | |  | |  | |  | |  |
| % age 18-29 |  |  | | |  | |  | |  | |  | |  |
| median income |  |  | | |  | |  | |  | |  | |  |
| % <highschool |  |  | | |  | |  | |  | |  | |  |
| % age 50-59 |  |  | | |  | |  | |  | |  | |  |
| % renter-occupied housing units |  |  | | |  | |  | |  | |  | |  |
| % unemployment |  |  | | |  | |  | |  | |  | |  |
| median value units built |  |  | | |  | |  | |  | |  | |  |
| % age 30-39 |  |  | | |  | |  | |  | |  | |  |
| % age >=60 |  |  | | |  | |  | |  | |  | |  |
| median year units built |  |  | | |  | |  | |  | |  | |  |
|  |  |  | | |  | |  | |  | |  | |  |
| S |  | 3.90445 | | |  | | 2.63730 | |  | | 1.97986 | |  |
| R-sq |  | 48.62% | | |  | | 76.58% | |  | | 86.82% | |  |
| R-sq(adj) |  | 48.46% | | |  | | 76.48% | |  | | 86.75% | |  |
| Mallows’ Cp |  | 12152.82 | | |  | | 5029.76 | |  | | 2424.14 | |  |
| AICc |  | 5273.37 | | |  | | 4531.26 | |  | | 3989.22 | |  |
| BIC |  | 5297.58 | | |  | | 4560.29 | |  | | 4023.08 | |  |
|  | **-----Step 4-----** | | | | **-----Step 5-----** | | | | **-----Step 6-----** | | | |  |
|  | **Coef** | | **P** | | **Coef** | | **P** | | **Coef** | | **P** | |  |
| Constant | 27.4290 | |  | | 27.4290 | |  | | 27.4290 | |  | |  |
| fast-food restaurant vf | 0.4997 | | 0.000 | | 0.4225 | | 0.000 | | 0.4281 | | 0.000 | |  |
| fitness and sports center vf | -1.1994 | | 0.000 | | -1.2640 | | 0.000 | | -1.2416 | | 0.000 | |  |
| nature park vf | -0.2339 | | 0.001 | | 0.0390 | | 0.545 | |  | |  | |  |
| % food stamp/SNAP | 1.8625 | | 0.000 | | 1.8377 | | 0.000 | | 1.8389 | | 0.000 | |  |
| % Asian | -2.0992 | | 0.000 | | -1.9273 | | 0.000 | | -1.9295 | | 0.000 | |  |
| % below poverty line | 1.4763 | | 0.000 | | 1.4222 | | 0.000 | | 1.4177 | | 0.000 | |  |
| % Black |  | |  | | 0.9013 | | 0.000 | | 0.8934 | | 0.000 | |  |
| % Hispanic or Latino |  | |  | |  | |  | |  | |  | |  |
| % age 18-29 |  | |  | |  | |  | |  | |  | |  |
| median income |  | |  | |  | |  | |  | |  | |  |
| % <highschool |  | |  | |  | |  | |  | |  | |  |
| % age 50-59 |  | |  | |  | |  | |  | |  | |  |
| % renter-occupied housing units |  | |  | |  | |  | |  | |  | |  |
| % unemployment |  | |  | |  | |  | |  | |  | |  |
| median value units built |  | |  | |  | |  | |  | |  | |  |
| % age 30-39 |  | |  | |  | |  | |  | |  | |  |
| % age >=60 |  | |  | |  | |  | |  | |  | |  |
| median year units built |  | |  | |  | |  | |  | |  | |  |
|  |  | |  | |  | |  | |  | |  | |  |
| S |  | | 1.71278 | |  | | 1.49679 | |  | | 1.49629 | |  |
| R-sq |  | | 90.14% | |  | | 92.48% | |  | | 92.48% | |  |
| R-sq(adj) |  | | 90.08% | |  | | 92.43% | |  | | 92.43% | |  |
| Mallows’ Cp |  | | 1578.31 | |  | | 984.83 | |  | | 983.58 | |  |
| AICc |  | | 3715.80 | |  | | 3461.53 | |  | | 3459.86 | |  |
| BIC |  | | 3754.47 | |  | | 3505.01 | |  | | 3498.53 | |  |
|  | **-----Step 7-----** | | | | **-----Step 8-----** | | | | **-----Step 9-----** | | | |  |
|  | **Coef** | | **P** | | **Coef** | | **P** | | **Coef** | | **P** | |  |
| Constant | 27.4290 | |  | | 27.4290 | |  | | 27.4290 | |  | |  |
| fast-food restaurant vf | 0.2099 | | 0.000 | | 0.1866 | | 0.000 | | 0.1779 | | 0.000 | |  |
| fitness and sports center vf | -0.3874 | | 0.000 | | -0.3487 | | 0.000 | | -0.1679 | | 0.010 | |  |
| nature park vf |  | |  | |  | |  | |  | |  | |  |
| % food stamp/SNAP | 1.1358 | | 0.000 | | 0.8742 | | 0.000 | | 0.8700 | | 0.000 | |  |
| % Asian | -1.5336 | | 0.000 | | -1.4321 | | 0.000 | | -1.4929 | | 0.000 | |  |
| % below poverty line | 1.1594 | | 0.000 | | 1.5864 | | 0.000 | | 1.4079 | | 0.000 | |  |
| % Black | 1.4482 | | 0.000 | | 1.4790 | | 0.000 | | 1.4129 | | 0.000 | |  |
| % Hispanic or Latino | 1.9308 | | 0.000 | | 2.0550 | | 0.000 | | 1.9595 | | 0.000 | |  |
| % age 18-29 |  | |  | | -0.6278 | | 0.000 | | -0.7182 | | 0.000 | |  |
| median income |  | |  | |  | |  | | -0.5206 | | 0.000 | |  |
| % <highschool |  | |  | |  | |  | |  | |  | |  |
| % age 50-59 |  | |  | |  | |  | |  | |  | |  |
| % renter-occupied housing units |  | |  | |  | |  | |  | |  | |  |
| % unemployment |  | |  | |  | |  | |  | |  | |  |
| median value units built |  | |  | |  | |  | |  | |  | |  |
| % age 30-39 |  | |  | |  | |  | |  | |  | |  |
| % age >=60 |  | |  | |  | |  | |  | |  | |  |
| median year units built |  | |  | |  | |  | |  | |  | |  |
|  |  | |  | |  | |  | |  | |  | |  |
| S |  | | 1.23843 | |  | | 1.12359 | |  | | 1.08823 | |  |
| R-sq |  | | 94.85% | |  | | 95.77% | |  | | 96.03% | |  |
| R-sq(adj) |  | | 94.81% | |  | | 95.73% | |  | | 96.00% | |  |
| Mallows’ Cp |  | | 380.53 | |  | | 149.42 | |  | | 83.53 | |  |
| AICc |  | | 3102.65 | |  | | 2919.37 | |  | | 2859.84 | |  |
| BIC |  | | 3146.14 | |  | | 2967.67 | |  | | 2912.95 | |  |
|  | **-----Step 10----** | | | | | **-----Step 11----** | | | | **-----Step 12----** | | | |
|  | **Coef** | | | **P** | | **Coef** | | **P** | | **Coef** | | **P** | |
| Constant | 27.4290 | | |  | | 27.4290 | |  | | 27.4290 | |  | |
| fast-food restaurant vf | 0.2142 | | | 0.000 | | 0.1859 | | 0.000 | | 0.2357 | | 0.000 | |
| fitness and sports center vf | -0.1782 | | | 0.005 | | -0.1585 | | 0.013 | | -0.2088 | | 0.002 | |
| nature park vf |  | | |  | |  | |  | |  | |  | |
| % food stamp/SNAP | 0.6761 | | | 0.000 | | 0.6848 | | 0.000 | | 0.6425 | | 0.000 | |
| % Asian | -1.5338 | | | 0.000 | | -1.5364 | | 0.000 | | -1.5513 | | 0.000 | |
| % below poverty line | 1.3037 | | | 0.000 | | 1.3132 | | 0.000 | | 1.3019 | | 0.000 | |
| % Black | 1.3829 | | | 0.000 | | 1.3741 | | 0.000 | | 1.3881 | | 0.000 | |
| % Hispanic or Latino | 1.399 | | | 0.000 | | 1.421 | | 0.000 | | 1.429 | | 0.000 | |
| % age 18-29 | -0.6731 | | | 0.000 | | -0.6255 | | 0.000 | | -0.6337 | | 0.000 | |
| median income | -0.5364 | | | 0.000 | | -0.5535 | | 0.000 | | -0.4094 | | 0.000 | |
| % <highschool | 0.776 | | | 0.000 | | 0.780 | | 0.000 | | 0.798 | | 0.000 | |
| % age 50-59 |  | | |  | | 0.1277 | | 0.003 | | 0.1491 | | 0.001 | |
| % renter-occupied housing units |  | | |  | |  | |  | | 0.1810 | | 0.005 | |
| % unemployment |  | | |  | |  | |  | |  | |  | |
| median value units built |  | | |  | |  | |  | |  | |  | |
| % age 30-39 |  | | |  | |  | |  | |  | |  | |
| % age >=60 |  | | |  | |  | |  | |  | |  | |
| median year units built |  | | |  | |  | |  | |  | |  | |
|  |  | | |  | |  | |  | |  | |  | |
| S |  | | | 1.06573 | |  | | 1.06137 | |  | | 1.05751 | |
| R-sq |  | | | 96.20% | |  | | 96.24% | |  | | 96.27% | |
| R-sq(adj) |  | | | 96.16% | |  | | 96.19% | |  | | 96.22% | |
| Mallows’ Cp |  | | | 43.14 | |  | | 36.21 | |  | | 30.23 | |
| AICc |  | | | 2821.31 | |  | | 2814.59 | |  | | 2808.74 | |
| BIC |  | | | 2879.22 | |  | | 2877.29 | |  | | 2876.24 | |
|  | **-----Step 13----** | | | | | **-----Step 14----** | | | | **-----Step 15----** | | | |
|  | **Coef** | | | **P** | | **Coef** | | **P** | | **Coef** | | **P** | |
| Constant | 27.4290 | | |  | | 27.4290 | |  | | 27.4290 | |  | |
| fast-food restaurant vf | 0.2338 | | | 0.000 | | 0.1998 | | 0.000 | | 0.1771 | | 0.000 | |
| fitness and sports center vf | -0.2033 | | | 0.002 | | -0.1623 | | 0.019 | | -0.1669 | | 0.016 | |
| nature park vf |  | | |  | |  | |  | |  | |  | |
| % food stamp/SNAP | 0.6307 | | | 0.000 | | 0.6160 | | 0.000 | | 0.6033 | | 0.000 | |
| % Asian | -1.5422 | | | 0.000 | | -1.5463 | | 0.000 | | -1.5581 | | 0.000 | |
| % below poverty line | 1.2732 | | | 0.000 | | 1.2844 | | 0.000 | | 1.2477 | | 0.000 | |
| % Black | 1.3858 | | | 0.000 | | 1.3872 | | 0.000 | | 1.3830 | | 0.000 | |
| % Hispanic or Latino | 1.459 | | | 0.000 | | 1.457 | | 0.000 | | 1.466 | | 0.000 | |
| % age 18-29 | -0.6419 | | | 0.000 | | -0.6546 | | 0.000 | | -0.6764 | | 0.000 | |
| median income | -0.3943 | | | 0.000 | | -0.3317 | | 0.000 | | -0.3300 | | 0.000 | |
| % <highschool | 0.789 | | | 0.000 | | 0.793 | | 0.000 | | 0.754 | | 0.000 | |
| % age 50-59 | 0.1443 | | | 0.001 | | 0.1482 | | 0.001 | | 0.1252 | | 0.005 | |
| % renter-occupied housing units | 0.1854 | | | 0.004 | | 0.2038 | | 0.002 | | 0.2769 | | 0.000 | |
| % unemployment | 0.0987 | | | 0.010 | | 0.0982 | | 0.010 | | 0.0963 | | 0.012 | |
| median value units built |  | | |  | | -0.1281 | | 0.052 | | -0.1573 | | 0.019 | |
| % age 30-39 |  | | |  | |  | |  | | -0.1033 | | 0.036 | |
| % age >=60 |  | | |  | |  | |  | |  | |  | |
| median year units built |  | | |  | |  | |  | |  | |  | |
|  |  | | |  | |  | |  | |  | |  | |
| S |  | | | 1.05432 | |  | | 1.05275 | |  | | 1.05083 | |
| R-sq |  | | | 96.29% | |  | | 96.31% | |  | | 96.33% | |
| R-sq(adj) |  | | | 96.24% | |  | | 96.25% | |  | | 96.27% | |
| Mallows’ Cp |  | | | 25.49 | |  | | 23.66 | |  | | 21.24 | |
| AICc |  | | | 2804.07 | |  | | 2802.29 | |  | | 2799.90 | |
| BIC |  | | | 2876.35 | |  | | 2879.36 | |  | | 2881.75 | |
|  | **-----Step 16----** | | | | | **-----Step 17----** | | | |  |  |  |  |
|  | **Coef** | | | **P** | | **Coef** | | **P** | |  |  |  |  |
| Constant | 27.4290 | | |  | | 27.4290 | |  | |  |  |  |  |
| fast-food restaurant vf | 0.1694 | | | 0.000 | | 0.1883 | | 0.000 | |  |  |  |  |
| fitness and sports center vf | -0.1741 | | | 0.012 | | -0.1539 | | 0.027 | |  |  |  |  |
| nature park vf |  | | |  | |  | |  | |  |  |  |  |
| % food stamp/SNAP | 0.5639 | | | 0.000 | | 0.5651 | | 0.000 | |  |  |  |  |
| % Asian | -1.5517 | | | 0.000 | | -1.5470 | | 0.000 | |  |  |  |  |
| % below poverty line | 1.2406 | | | 0.000 | | 1.2495 | | 0.000 | |  |  |  |  |
| % Black | 1.3767 | | | 0.000 | | 1.3580 | | 0.000 | |  |  |  |  |
| % Hispanic or Latino | 1.387 | | | 0.000 | | 1.364 | | 0.000 | |  |  |  |  |
| % age 18-29 | -0.7345 | | | 0.000 | | -0.7327 | | 0.000 | |  |  |  |  |
| median income | -0.3608 | | | 0.000 | | -0.3274 | | 0.000 | |  |  |  |  |
| % <highschool | 0.779 | | | 0.000 | | 0.759 | | 0.000 | |  |  |  |  |
| % age 50-59 | 0.1152 | | | 0.011 | | 0.1060 | | 0.019 | |  |  |  |  |
| % renter-occupied housing units | 0.2487 | | | 0.001 | | 0.2770 | | 0.000 | |  |  |  |  |
| % unemployment | 0.0969 | | | 0.011 | | 0.0953 | | 0.012 | |  |  |  |  |
| median value units built | -0.1477 | | | 0.028 | | -0.1936 | | 0.006 | |  |  |  |  |
| % age 30-39 | -0.1524 | | | 0.005 | | -0.1576 | | 0.003 | |  |  |  |  |
| % age >=60 | -0.1426 | | | 0.025 | | -0.1457 | | 0.022 | |  |  |  |  |
| median year units built |  | | |  | | -0.0845 | | 0.048 | |  |  |  |  |
|  |  | | |  | |  | |  | |  |  |  |  |
| S |  | | | 1.04856 | |  | | 1.04692 | |  |  |  |  |
| R-sq |  | | | 96.35% | |  | | 96.36% | |  |  |  |  |
| R-sq(adj) |  | | | 96.28% | |  | | 96.29% | |  |  |  |  |
| Mallows’ Cp |  | | | 18.18 | |  | | 16.28 | |  |  |  |  |
| AICc |  | | | 2796.85 | |  | | 2794.96 | |  |  |  |  |
| BIC |  | | | 2883.48 | |  | | 2886.35 | |  |  |  |  |
| α to enter = 0.15, α to remove = 0.15 | | | | | | | | | |  |  |  |  |

Table S9. Stepwise regression result of Buffalo.

|  | **-----Step 1----** | | | | | **-----Step 2----** | | | | | | **-----Step 3----** | | | | | |
| --- | --- | --- | --- | --- | --- | --- | --- | --- | --- | --- | --- | --- | --- | --- | --- | --- | --- |
|  | **Coef** | | **P** | | | **Coef** | | | **P** | | | **Coef** | | | | **P** | |
| Constant | | | 37.695 | | |  | | | 37.695 | | |  | | | 37.695 |  | |
| fast-food restaurant vf | | | 1.505 | | | 0.018 | | | 0.624 | | | 0.155 | | | 0.431 | 0.298 | |
| fitness and sports center vf | | | -4.278 | | | 0.000 | | | -2.027 | | | 0.000 | | | -2.114 | 0.000 | |
| nature park vf | | | -1.373 | | | 0.037 | | | -0.592 | | | 0.191 | | |  |  | |
| % food stamp/SNAP | | |  | | |  | | | 4.534 | | | 0.000 | | | 4.656 | 0.000 | |
| % Black | | |  | | |  | | |  | | |  | | |  |  | |
| % age 18-29 | | |  | | |  | | |  | | |  | | |  |  | |
| % below poverty line | | |  | | |  | | |  | | |  | | |  |  | |
| median value units built | | |  | | |  | | |  | | |  | | |  |  | |
| population density | | |  | | |  | | |  | | |  | | |  |  | |
| % age 30-39 | | |  | | |  | | |  | | |  | | |  |  | |
| median income | | |  | | |  | | |  | | |  | | |  |  | |
| % <highschool | | |  | | |  | | |  | | |  | | |  |  | |
| % age >=60 | | |  | | |  | | |  | | |  | | |  |  | |
| % married | | |  | | |  | | |  | | |  | | |  |  | |
|  | | |  | | |  | | |  | | |  | | |  |  | |
| S | | |  | | | 5.15181 | | |  | | | 3.50779 | | |  | 3.52564 | |
| R-sq | | |  | | | 47.98% | | |  | | | 76.22% | | |  | 75.64% | |
| R-sq(adj) | | |  | | | 45.85% | | |  | | | 74.89% | | |  | 74.64% | |
| Mallows’ Cp | | |  | | | 1055.68 | | |  | | | 447.26 | | |  | 457.73 | |
| AICc | | |  | | | 477.71 | | |  | | | 419.81 | | |  | 419.30 | |
| BIC | | |  | | | 488.59 | | |  | | | 432.68 | | |  | 430.18 | |
|  | **-----Step 4----** | | | | | **-----Step 5----** | | | | | | **-----Step 6----** | | | | | |
|  | **Coef** | | **P** | | | **Coef** | | | **P** | | | **Coef** | | | | **P** | |
| Constant | 37.695 | |  | | | 37.695 | | |  | | | 37.695 | | | |  | |
| fast-food restaurant vf |  | |  | | |  | | |  | | |  | | | |  | |
| fitness and sports center vf | -2.038 | | 0.000 | | | -0.767 | | | 0.021 | | | -0.652 | | | | 0.021 | |
| nature park vf |  | |  | | |  | | |  | | |  | | | |  | |
| % food stamp/SNAP | 4.741 | | 0.000 | | | 3.799 | | | 0.000 | | | 3.519 | | | | 0.000 | |
| % Black |  | |  | | | 3.329 | | | 0.000 | | | 3.231 | | | | 0.000 | |
| % age 18-29 |  | |  | | |  | | |  | | | -1.269 | | | | 0.000 | |
| % below poverty line |  | |  | | |  | | |  | | |  | | | |  | |
| median value units built |  | |  | | |  | | |  | | |  | | | |  | |
| population density |  | |  | | |  | | |  | | |  | | | |  | |
| % age 30-39 |  | |  | | |  | | |  | | |  | | | |  | |
| median income |  | |  | | |  | | |  | | |  | | | |  | |
| % <highschool |  | |  | | |  | | |  | | |  | | | |  | |
| % age >=60 |  | |  | | |  | | |  | | |  | | | |  | |
| % married |  | |  | | |  | | |  | | |  | | | |  | |
|  |  | |  | | |  | | |  | | |  | | | |  | |
| S |  | | 3.52802 | | |  | | | 2.23782 | | |  | | | | 1.88738 | |
| R-sq |  | | 75.27% | | |  | | | 90.19% | | |  | | | | 93.11% | |
| R-sq(adj) |  | | 74.60% | | |  | | | 89.78% | | |  | | | | 92.73% | |
| Mallows’ Cp |  | | 463.66 | | |  | | | 143.21 | | |  | | | | 81.88 | |
| AICc |  | | 418.17 | | |  | | | 349.30 | | |  | | | | 324.37 | |
| BIC |  | | 426.99 | | |  | | | 360.18 | | |  | | | | 337.23 | |
|  | **-----Step 7----** | | | | | **-----Step 8----** | | | | | | **-----Step 9----** | | | | | |
|  | **Coef** | | **P** | | | **Coef** | | | **P** | | | **Coef** | | | | **P** | |
| Constant | 37.695 | |  | | | 37.695 | | |  | | | 37.695 | | | |  | |
| fast-food restaurant vf |  | |  | | |  | | |  | | |  | | | |  | |
| fitness and sports center vf | -0.432 | | 0.071 | | | -0.322 | | | 0.159 | | |  | | | |  | |
| nature park vf |  | |  | | |  | | |  | | |  | | | |  | |
| % food stamp/SNAP | 1.806 | | 0.000 | | | 1.540 | | | 0.000 | | | 1.554 | | | | 0.000 | |
| % Black | 3.459 | | 0.000 | | | 3.424 | | | 0.000 | | | 3.538 | | | | 0.000 | |
| % age 18-29 | -1.584 | | 0.000 | | | -1.420 | | | 0.000 | | | -1.439 | | | | 0.000 | |
| % below poverty line | 1.947 | | 0.000 | | | 1.872 | | | 0.000 | | | 1.946 | | | | 0.000 | |
| median value units built |  | |  | | | -0.719 | | | 0.003 | | | -0.773 | | | | 0.002 | |
| population density |  | |  | | |  | | |  | | |  | | | |  | |
| % age 30-39 |  | |  | | |  | | |  | | |  | | | |  | |
| median income |  | |  | | |  | | |  | | |  | | | |  | |
| % <highschool |  | |  | | |  | | |  | | |  | | | |  | |
| % age >=60 |  | |  | | |  | | |  | | |  | | | |  | |
| % married |  | |  | | |  | | |  | | |  | | | |  | |
|  |  | |  | | |  | | |  | | |  | | | |  | |
| S |  | | 1.58791 | | |  | | | 1.50385 | | |  | | | | 1.51473 | |
| R-sq |  | | 95.19% | | |  | | | 95.75% | | |  | | | | 95.63% | |
| R-sq(adj) |  | | 94.86% | | |  | | | 95.39% | | |  | | | | 95.32% | |
| Mallows’ Cp |  | | 38.92 | | |  | | | 28.90 | | |  | | | | 29.56 | |
| AICc |  | | 299.11 | | |  | | | 292.13 | | |  | | | | 291.84 | |
| BIC |  | | 313.89 | | |  | | | 308.76 | | |  | | | | 306.62 | |
|  | **----Step 10----** | | | | | | **----Step 11----** | | | | | | **----Step 12----** | | | |  |
|  | **Coef** | | | **P** | | | **Coef** | | | **P** | | | **Coef** | | **P** | |  |
| Constant | 37.695 | | |  | | | 37.695 | | |  | | | 37.695 | |  | |  |
| fast-food restaurant vf |  | | |  | | |  | | |  | | |  | |  | |  |
| fitness and sports center vf |  | | |  | | |  | | |  | | |  | |  | |  |
| nature park vf |  | | |  | | |  | | |  | | |  | |  | |  |
| % food stamp/SNAP | 1.361 | | | 0.000 | | | 1.369 | | | 0.000 | | | 0.926 | | 0.012 | |  |
| % Black | 3.510 | | | 0.000 | | | 3.735 | | | 0.000 | | | 3.638 | | 0.000 | |  |
| % age 18-29 | -1.371 | | | 0.000 | | | -1.292 | | | 0.000 | | | -1.311 | | 0.000 | |  |
| % below poverty line | 2.088 | | | 0.000 | | | 2.072 | | | 0.000 | | | 1.767 | | 0.000 | |  |
| median value units built | -0.786 | | | 0.001 | | | -0.913 | | | 0.000 | | | -0.808 | | 0.000 | |  |
| population density | -0.609 | | | 0.000 | | | -0.694 | | | 0.000 | | | -0.640 | | 0.000 | |  |
| % age 30-39 |  | | |  | | | 0.544 | | | 0.004 | | | 0.600 | | 0.001 | |  |
| median income |  | | |  | | |  | | |  | | | -0.947 | | 0.009 | |  |
| % <highschool |  | | |  | | |  | | |  | | |  | |  | |  |
| % age >=60 |  | | |  | | |  | | |  | | |  | |  | |  |
| % married |  | | |  | | |  | | |  | | |  | |  | |  |
|  |  | | |  | | |  | | |  | | |  | |  | |  |
| S |  | | | 1.39673 | | |  | | | 1.32399 | | |  | | 1.26716 | |  |
| R-sq |  | | | 96.33% | | |  | | | 96.75% | | |  | | 97.07% | |  |
| R-sq(adj) |  | | | 96.02% | | |  | | | 96.42% | | |  | | 96.72% | |  |
| Mallows’ Cp |  | | | 16.27 | | |  | | | 9.21 | | |  | | 4.38 | |  |
| AICc |  | | | 280.75 | | |  | | | 273.98 | | |  | | 268.74 | |  |
| BIC |  | | | 297.38 | | |  | | | 292.38 | | |  | | 288.85 | |  |
|  | **----Step 13----** | | | | | | **----Step 14----** | | | | | | **----Step 15----** | | | |  |
|  | **Coef** | | | **P** | | | **Coef** | | | **P** | | | **Coef** | | **P** | |  |
| Constant | 37.695 | | |  | | | 37.695 | | |  | | | 37.695 | |  | |  |
| fast-food restaurant vf |  | | |  | | |  | | |  | | |  | |  | |  |
| fitness and sports center vf |  | | |  | | |  | | |  | | |  | |  | |  |
| nature park vf |  | | |  | | |  | | |  | | |  | |  | |  |
| % food stamp/SNAP | 0.580 | | | 0.153 | | |  | | |  | | |  | |  | |  |
| % Black | 3.757 | | | 0.000 | | | 3.838 | | | 0.000 | | | 3.899 | | 0.000 | |  |
| % age 18-29 | -1.282 | | | 0.000 | | | -1.317 | | | 0.000 | | | -1.500 | | 0.000 | |  |
| % below poverty line | 1.755 | | | 0.000 | | | 1.930 | | | 0.000 | | | 1.656 | | 0.000 | |  |
| median value units built | -0.780 | | | 0.000 | | | -0.800 | | | 0.000 | | | -0.640 | | 0.004 | |  |
| population density | -0.629 | | | 0.000 | | | -0.638 | | | 0.000 | | | -0.703 | | 0.000 | |  |
| % age 30-39 | 0.567 | | | 0.002 | | | 0.565 | | | 0.002 | | | 0.403 | | 0.040 | |  |
| median income | -0.884 | | | 0.013 | | | -1.059 | | | 0.002 | | | -1.182 | | 0.001 | |  |
| % <highschool | 0.467 | | | 0.076 | | | 0.646 | | | 0.006 | | | 0.652 | | 0.005 | |  |
| % age >=60 |  | | |  | | |  | | |  | | | -0.435 | | 0.063 | |  |
| % married |  | | |  | | |  | | |  | | |  | |  | |  |
|  |  | | |  | | |  | | |  | | |  | |  | |  |
| S |  | | | 1.24667 | | |  | | | 1.25663 | | |  | | 1.23340 | |  |
| R-sq |  | | | 97.20% | | |  | | | 97.12% | | |  | | 97.26% | |  |
| R-sq(adj) |  | | | 96.83% | | |  | | | 96.78% | | |  | | 96.90% | |  |
| Mallows’ Cp |  | | | 3.45 | | |  | | | 3.33 | | |  | | 2.17 | |  |
| AICc |  | | | 267.82 | | |  | | | 267.46 | | |  | | 266.17 | |  |
| BIC |  | | | 289.54 | | |  | | | 287.56 | | |  | | 287.89 | |  |
|  | **----Step 16----** | | | | | |  |  |  |  |  |  |  |  |  |  |  |
|  | **Coef** | | | **P** | | |  |  |  |  |  |  |  |  |  |  |  |
| Constant | 37.695 | | |  | | |  |  |  |  |  |  |  |  |  |  |  |
| fast-food restaurant vf |  | | |  | | |  |  |  |  |  |  |  |  |  |  |  |
| fitness and sports center vf |  | | |  | | |  |  |  |  |  |  |  |  |  |  |  |
| nature park vf |  | | |  | | |  |  |  |  |  |  |  |  |  |  |  |
| % food stamp/SNAP |  | | |  | | |  |  |  |  |  |  |  |  |  |  |  |
| % Black | 3.771 | | | 0.000 | | |  |  |  |  |  |  |  |  |  |  |  |
| % age 18-29 | -1.763 | | | 0.000 | | |  |  |  |  |  |  |  |  |  |  |  |
| % below poverty line | 1.569 | | | 0.000 | | |  |  |  |  |  |  |  |  |  |  |  |
| median value units built | -0.662 | | | 0.003 | | |  |  |  |  |  |  |  |  |  |  |  |
| population density | -0.735 | | | 0.000 | | |  |  |  |  |  |  |  |  |  |  |  |
| % age 30-39 | 0.343 | | | 0.079 | | |  |  |  |  |  |  |  |  |  |  |  |
| median income | -1.006 | | | 0.004 | | |  |  |  |  |  |  |  |  |  |  |  |
| % <highschool | 0.684 | | | 0.003 | | |  |  |  |  |  |  |  |  |  |  |  |
| % age >=60 | -0.553 | | | 0.022 | | |  |  |  |  |  |  |  |  |  |  |  |
| % married | -0.397 | | | 0.084 | | |  |  |  |  |  |  |  |  |  |  |  |
|  |  | | |  | | |  |  |  |  |  |  |  |  |  |  |  |
| S |  | | | 1.21475 | | |  |  |  |  |  |  |  |  |  |  |  |
| R-sq |  | | | 97.39% | | |  |  |  |  |  |  |  |  |  |  |  |
| R-sq(adj) |  | | | 96.99% | | |  |  |  |  |  |  |  |  |  |  |  |
| Mallows’ Cp |  | | | 1.53 | | |  |  |  |  |  |  |  |  |  |  |  |
| AICc |  | | | 265.48 | | |  |  |  |  |  |  |  |  |  |  |  |
| BIC |  | | | 288.73 | | |  |  |  |  |  |  |  |  |  |  |  |

α to enter = 0.15, α to remove = 0.15
